# Lipid Nanoparticle Co‐Delivery of mRNA and a Small Molecule Drug for Oral Cancer Chemoimmunotherapy

**DOI:** 10.1002/adma.73721

**Published:** 2026-07-14

**Authors:** Marshall S. Padilla, Jacqueline J. Li, Qunzhou Zhang, Korey Patwari, Shihong Shi, Hannah M. Yamagata, Ryann A. Joseph, Briyanna N. Hymms, Sridatta V. Teerdhala, Emily Fitzgerald, Ori Z. Chalom, Kushol Gupta, Mohamad‐Gabriel Alameh, Drew Weissman, Anh D. Le, Michael J. Mitchell

**Affiliations:** ^1^ Department of Bioengineering School of Engineering and Applied Science University of Pennsylvania Philadelphia Pennsylvania USA; ^2^ Department of Oral and Maxillofacial Surgery and Pharmacology University of Pennsylvania School of Dental Medicine Philadelphia Pennsylvania USA; ^3^ Department of Biochemistry and Biophysics Perelman School of Medicine University of Pennsylvania Philadelphia Pennsylvania USA; ^4^ Penn Institute for RNA Innovation Perelman School of Medicine University of Pennsylvania Philadelphia Pennsylvania USA; ^5^ Department of Medicine University of Pennsylvania Philadelphia Pennsylvania USA; ^6^ Department of Oral & Maxillofacial Surgery Penn Medicine Hospital of the University of Pennsylvania Philadelphia Pennsylvania USA; ^7^ Abramson Cancer Center Perelman School of Medicine University of Pennsylvania Philadelphia Pennsylvania USA; ^8^ Institute for Immunology Perelman School of Medicine University of Pennsylvania Philadelphia Pennsylvania USA; ^9^ Cardiovascular Institute Perelman School of Medicine University of Pennsylvania Philadelphia Pennsylvania USA; ^10^ Institute for Regenerative Medicine Perelman School of Medicine University of Pennsylvania Philadelphia Pennsylvania USA; ^11^ Center for Precision Engineering for Health University of Pennsylvania Philadelphia Pennsylvania USA

**Keywords:** lipid nanoparticles, mRNA therapy, oral squamous cell carcinoma, tumor‐associated macrophages

## Abstract

Oral squamous cell carcinoma (OSCC) represents 90% of all head and neck cancers. Despite decades of research, the 5‐year survival rate is 50%. Strikingly, the overall incidence rate is projected to increase by 30% in the next 10 years, which may result in a sharp increase in mortality. Two fundamental aspects of OSCC are that it progresses via the inactivation and mutation of tumor suppressor genes (TSGs) and has a “cold” tumor microenvironment (TME). A major barrier in the treatment of OSCC is the lack of novel therapies clinicians have at their disposal that are designed to disrupt tumor progression by reshaping the cold tumors into inflammatory “hot” tumors. To overcome these obstacles, we employed a lipid nanoparticle (LNP) that co‐encapsulates p53 mRNA and the small molecule ciclopirox (CPX). We demonstrate that both drugs have innate chemotherapeutic properties by facilitating caspase activation. Moreover, these therapies can create a less immunosuppressive TME in part by repolarizing tumor‐associated macrophages (TAMs) to M1‐like phenotypes. When formulated together, our platform provides an all‐in‐one approach for OSCC, effective in both p53‐therapy‐susceptible and p53‐therapy‐resistant models. Additionally, this work offers a template for a delivery platform capable of tackling multiple mechanisms of OSCC progression and survival.

## Graphical abstract

Co‐encapsulation of p53 mRNA and the small molecule ciclopirox within a lipid nanoparticle yields an all‐in‐one chemoimmunotherapy for oral squamous cell carcinoma. The platform engages caspase‐driven apoptosis in cancer cells while repolarizing tumor‐associated macrophages, achieving tumor reduction in both p53‐susceptible and p53‐resistant models and overcoming a key limitation of prior monotherapies.

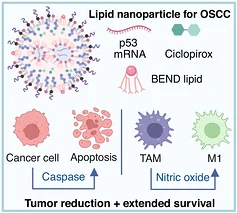

## 1 Introduction

Head and neck squamous cell carcinoma (HNSCC) is the sixth most common form of cancer in the world. Approximately 450 000 people die from this disease each year, with oral squamous cell carcinoma (OSCC) representing over 90% of HNSCC [1]. Unfortunately, the incidence rate of OSCC is projected to increase by 30% by the end of the decade. Standard of care for OSCC involves ablative surgery and radiation, and although these therapies can be curative, up to 50% of patients experience significant functional morbidity, including difficulty speaking and swallowing. For patients with recurrent or metastatic disease, treatment options remain limited. Cetuximab, an anti‐EGFR monoclonal antibody, was the first targeted therapy approved for HNSCC; however, its efficacy in oral cavity cancers is lower than in other head and neck subsites, with response rates for recurrent/metastatic OSCC of only 37%–57% when combined with radiation or chemotherapy and a median overall survival of 8–14 months [2]. More recently, immune checkpoint inhibitors have reshaped the treatment landscape, with nivolumab and pembrolizumab receiving FDA approval for recurrent/metastatic HNSCC based on the CheckMate‐141 [3] and KEYNOTE‐048 [4] trials, respectively. Yet, overall response rates for anti‐PD‐1 monotherapy remain below 20% in unselected HNSCC populations, and even in PD‐L1‐high OSCC patients, durable responses are achieved in only a subset of individuals [5]. Combined with decades‐old platinum‐based chemotherapies such as cisplatin that remain the backbone of systemic treatment, these modest response rates underscore the urgent need for novel therapeutic strategies for OSCC. This includes the large population of tumor‐associated macrophages (TAMs) in the oral tumor microenvironment (TME) that strongly contribute to cancer immune evasion. Additionally, oral tumors have a high mutational rate of intrinsic tumor suppressor genes (TSGs), including *TP53*, that leads to their inactivation and contributes to tumor development and progression through regulating DNA repair, cell proliferation, apoptosis, and even the cancer immune response [6, 7].

To address the multitude of mechanisms that cause cancer progression, it has become more common to co‐administer therapeutics that target different cancer axes [8, 9]. While effective, it can lead to severe toxicity for the patient due to the combined side effects of each drug. Co‐formulation into a single drug delivery vehicle represents an exciting alternative as both therapeutics can be simultaneously loaded and administered. Drug delivery vehicles also offer cargo protection and can be tuned to target tumors directly, limiting off‐target accumulation [10]. Still, if the therapeutics have different chemical characteristics, such as water solubilities, efficient loading can be difficult. Recently, lipid nanoparticles (LNPs) have emerged as the preeminent vehicle for the delivery of RNAs, being the FDA‐approved carriers for the Pfizer/BioNTech and Moderna COVID‐19 mRNA vaccines [11]. LNPs have aqueous and lipid microenvironments, allowing for the encapsulation of hydrophilic and lipophilic drugs. For example, we have demonstrated that LNPs can be co‐loaded with Bcl‐2 siRNA and doxorubicin for synergistic cancer therapy [12]. Even though there has been research in optimizing LNPs for OSCC, there are no studies on the co‐delivery of multiple therapeutics with LNPs for OSCC therapy [13, 14].

Here, we construct an LNP platform that co‐encapsulates RNA and a small‐molecule drug to reduce tumor viability and polarize the TME. For the RNA component, we deliver p53 mRNA as p53 is underexpressed or mutated in over 70% of OSCC cases [15]. Several p53 restoration therapies have been developed for hepatocarcinoma and non‐small cell lung cancer; however, no p53 mRNA therapies have been utilized for OSCC [16, 17]. p53 expression mediates apoptosis in cancer cells, and recent studies have found that increased p53 expression can halt M2‐like polarization in macrophages [18]. However, p53 therapies have failed in clinical trials due to mediocre performance and the toxicity of viral vector delivery vehicles that are the current standard for tumor suppressor replacement therapy. Although OSCC tumors can be broadly classified as p53‐deficient, the cell populations have heterogeneous expression of wild‐type p53 and mutated p53 species. Thus, the purpose of this study is to co‐deliver p53 mRNA and ciclopirox (CPX), an FDA‐approved anti‐fungal medication that has been repurposed as a chemotherapeutic in bladder cancer, pancreatic cancer, and colorectal cancer in preclinical studies [19, 20].

**FIGURE 1:**
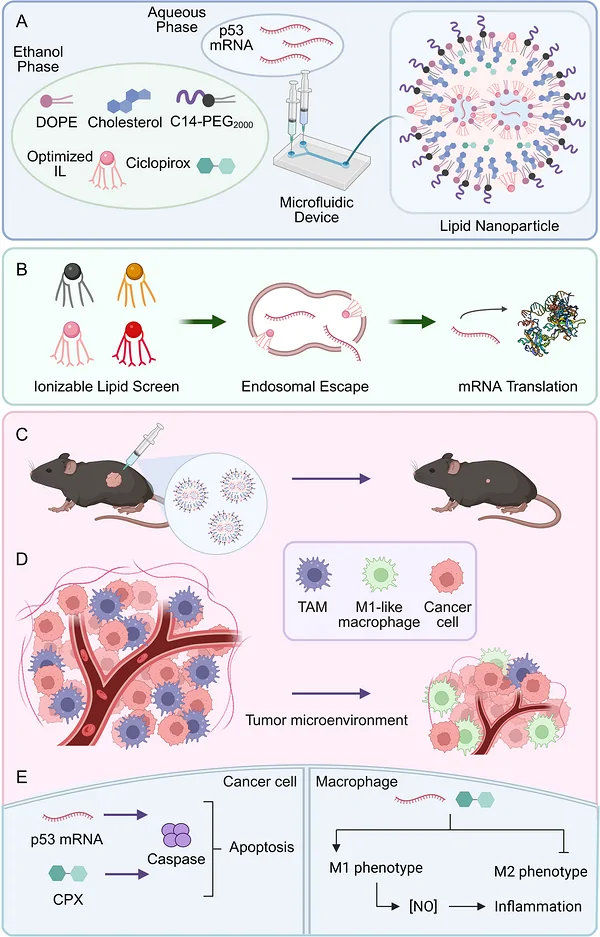
Overview of the lipid nanoparticle (LNP) co‐delivery platform. (A), LNPs are formulated via a microfluidic device, combining an aqueous mRNA phase with an ethanolic phase containing the lipid excipients. (B), LNPs are screened with different ionizable lipids to identify a top performer. Ionizable lipids aid in endosomal escape by disrupting endosomal bilayers, releasing RNA into the cytosol where it is translated into protein. (C–D), LNP treatment delays tumor growth by (C) killing tumor cells and (D) altering the TME via polarization of TAMs to M1‐like macrophages. (E), Cancer cell killing is mediated by caspase pathways, while macrophage polarization involves the expression of M1‐like markers and increased nitric oxide production.

Both drugs require a drug delivery carrier, as CPX has poor water solubility and p53 mRNA cannot cross cell membranes due to its large size, negative charge, and susceptibility to degradation by nucleases [21]. To achieve potent delivery of both drugs, we co‐encapsulated them into a single LNP formulation (Figure 1A). CPX is soluble in ethanol and thus can be incorporated into the ethanolic phase of the LNP formulation process that also contains the other lipid excipients. The resulting LNP facilitated cell killing in human and murine OSCC lines, which we determined to be a result of increased caspase activity.

Moreover, we found that both p53 and CPX can repolarize TAMs to a more M1‐like phenotype in OSCC, although the relative effect of each therapeutic depends on the cell type (Figure 1C–E). We then demonstrate that the combination therapy allows for the reduction of tumor burden in models where one therapy is ineffective. This highlights that this platform can be implemented for a wider range of OSCC tumors in place of traditional monotherapies.

## 2 Results

### 2.1 Optimization of a Potent Lipid Nanoparticle for Oral Squamous Cell Carcinoma

LNPs are formulated using an ethanol phase that dissolves lipid components and a citrate phase that contains the mRNA. The lipids include a phospholipid, cholesterol, PEGylated lipid, and an ionizable lipid. The ionizable lipid is central to the efficacy of the LNP due to its structure that amalgamates amines and hydrophobic tails. Amines allow for switchable cationic charge, whereas the tails endow the lipid with hydrophobicity. As such, ionizable lipids protonate in the citrate buffer, allowing the molecules to electrostatically bind to negatively charged mRNA, enwrapping it in the hydrophobic tails, and enabling it to be encapsulated in the LNP. After formulation, the LNPs are dialyzed against phosphate buffer saline (PBS) to neutralize the charge, as particles with permanent positive charges can induce toxicity [22]. Once LNPs enter cells, they proceed through the low pH endo‐lysosomal pathway, allowing the ionizable lipids to regain their positive charge. The positively charged ionizable lipids then disrupt the endosomes and lysosomes and facilitate mRNA escape into the cytosol to begin translation [23]. Since ionizable lipids play a pivotal role in LNP delivery, it is important to tailor their structure as different lipids are optimal for certain biological environments [24, 25].

**FIGURE 2:**
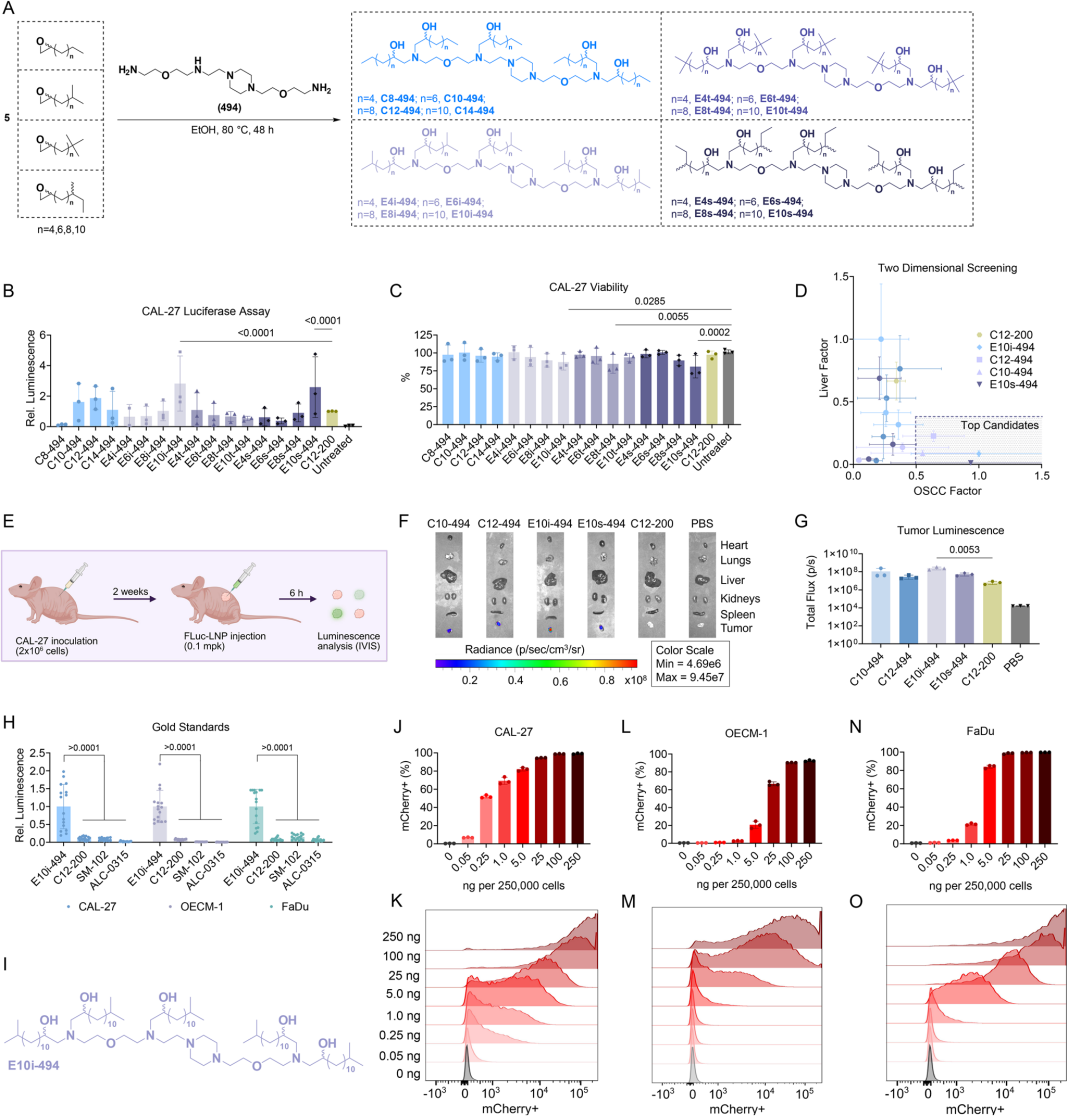
Optimization of LNPs for oral cancer. (A), Ionizable lipids were synthesized via an S_N_2 reaction between a linear or terminally branched alkyl epoxide and the polyamine, 494. (B–C), CAL‐27 cells were transfected with FLuc‐containing LNPs at a dose of 20 ng of mRNA per 20 000 cells. After 24 h, (B) luminescence and (C) viability were measured. Relative luminescence and viability are reported as mean ± SD of *n* = 3 biological replicates, with each biological replicate consisting of *n* = 4 technical replicates. (D), CAL‐27 cell luminescence (*x*‐axis) was plotted against liver luminescence after intravenous administration in C57BL/6J mice with LNPs containing FLuc mRNA at a dose of 0.1 mpk (*y*‐axis). The liver luminescence data were previously obtained [26]. (E), Nu/J mice were inoculated with two million CAL‐27 cells on the right flank, and after 2 weeks, LNPs were intratumorally administered at 0.1 mpk. (F–G), After 6 h, the mice were sacrificed, and organ luminescence images were (F) captured and (G) quantified utilizing IVIS. Total flux is reported as mean ± SD of *n* = 3. (H), The top ionizable lipid performer, E10i‐494, and three gold standard lipids, C12‐200, SM‐102, and ALC‐0315, loaded with FLuc mRNA were dosed in CAL‐27, OECM‐1, and FaDu cells at 20 ng of mRNA per 20 000 cells. After 24 h, luminescence was quantified. Relative luminescence is reported as mean ± SD of *n* = 16 replicates. (I), Structure of E10i‐494 ionizable lipid. (J–O), E10i‐494 was reformulated with mCherry mRNA and transfected in CAL‐27, OECM‐1, and FaDu cells at increasing dosages per 250 000 cells. After 24 h, the oral cancer cells were analyzed by flow cytometry to measure (J) mCherry+ CAL‐27 cells, (K) CAL‐27 fluorescence intensity, (L) mCherry+ OECM‐1 cells, (M) OECM‐1 fluorescence intensity, (N) mCherry+ FaDu cells, and (O) FaDu fluorescence intensity. mCherry positivity is reported as mean ± SD of *n* = 3. Representative data were used for the fluorescence intensity plots. (B–C), Nested one‐way ANOVA with post hoc Holm–Šídák correction for multiple comparisons was used to compare (B) luminescence against C12‐200 and (C) viability against untreated cells. (G), One‐way ANOVA with post hoc Holm–Šídák correction for multiple comparisons was used to compare tumor luminescence to C12‐200. (H), Two‐way ANOVA with post hoc Holm–Šídák correction for multiple comparisons was used to compare E10i‐494 relative luminescence against individual gold standards for each cell type.

We have recently developed a class of ionizable lipids with terminal branching that enhance endosomal escape, leading to more efficacious mRNA delivery [26]. These Branched ENdosomal Disruptor (BEND) lipids were synthesized by reacting a linear or branched alkyl epoxide with a polyamine core via an S_N_2 reaction (Figure 2A and Figure S1). Each ionizable lipid varied in its total alkyl length as well as branching type, with the latter including non‐branched or linear, isopropyl, tert‐butyl, and sec‐butyl structures, with sixteen ionizable lipids being synthesized in total. The ionizable lipids were then formulated into LNPs with firefly luciferase (FLuc) mRNA using a molar ratio of 35:16:46.5:2.5 for the ionizable lipid, 1,2‐dioleoyl‐sn‐glycero‐3‐phosphoethanolamine (DOPE), cholesterol, and 14:0 PEG2000 PE, respectively. LNPs were prepared at a 10:1 ionizable lipid to mRNA weight ratio using herringbone microfluidic devices [27]. Each of the ionizable lipids was formulated into separate LNPs, with the gold standard, C12‐200, prepared as a positive control for a total of seventeen unique LNP formulations. Traditionally, in vitro assays are used to screen LNPs for in vivo applications; however, transfection trends in 2D monolayers often do not correlate with in vivo trends [28]. Instead, we developed a multi‐tiered screening approach to identify a lead LNP candidate that has high transfection in oral cancer cells and low off‐target mRNA delivery in the liver. For the first step, the LNPs were incubated in CAL‐27 cells, a common human tongue squamous cell carcinoma line, at a dose of 20 ng of encapsulated mRNA per 20 000 cells. After 24 h, a luminescence assay (Figure 2B) and toxicity assay (Figure 2C) were performed to evaluate the efficacy and safety of the LNPs in vitro, respectively. Two LNPs, E10i‐494 and E10s‐494, had a 2‐fold increase in luciferase expression compared to C12‐200, while all LNPs maintained cell viability above 75%. Utilizing previous data on intravenous liver delivery of BEND LNPs, we plotted the liver luminescence of each LNP against its transfection in CAL‐27 cells and identified four LNPs, C10‐494, C12‐494, E10i‐494, and E10s‐494 with low hepatic tropism and potent OSCC delivery (Figure 2D). Since LNPs have naturally high accumulation in the liver, identifying formulations with low liver transfection is important for decreasing off‐target toxicity. We then created a subcutaneous xenograft tumor model using CAL‐27 cells in Nu/J mice. After 2 weeks, we intratumorally administered the top four LNPs along with C12‐200 at a dose of 0.1 mpk (mg of encapsulated mRNA per kg of body weight) (Figure 2E). After 6 h, the mice received intraperitoneal injections of D‐luciferin and were sacrificed. The heart, lungs, liver, kidneys, spleen, and tumor were dissected and imaged for luminescence by an In Vivo Imaging System (IVIS) (Figure 2F). E10i‐494 emerged as the lead performer, having 50‐fold higher tumor luminescence compared to the gold standard C12‐200 (Figure 2G).

To verify the potency of E10i‐494, we compared it against lead gold standard lipids in additional cell lines. E10i‐494 was evaluated in CAL‐27 cells as well as OECM‐1 and FaDu cells—other human OSCC lines—against C12‐200, SM‐102, and ALC‐0315. SM‐102 and ALC‐0315 are the LNPs based on the mRNA COVID‐19 vaccine formulations for Moderna and Pfizer/BioNTech, respectively. When dosed at 20 ng per 20 000 cells, E10i‐494 outperformed all gold standards in all three cell lines by 2‐ to 5‐fold (Figure 2H). We hypothesize that the strong ability to transfect the OSCC lines is due to the isopropyl terminal branching at the end of the ionizable lipid tails, which we found to aid in mRNA endosomal escape (Figure 2I). To further probe potency, we reformulated E10i‐494 with mCherry mRNA and performed a dose escalation study in all three cell lines, ranging from 0.05 to 250 ng per 250 000 cells. Differences were observed in all cell lines, with CAL‐27 being the easiest to transfect, as a 0.25 ng dose resulted in 50% cell transfection while 80% of cells were transfected at a 5 ng dose (Figure 2J,K). Meanwhile, OECM‐1 cells were the most difficult to transfect, as a 25 ng dose only resulted in 60% transfection (Figure 2L,M). Lastly, FaDu cells had a medium transfectability, with 1 ng inducing 20% positivity while 5 ng resulted in 80% positivity (Figure 2N,O).

### 2.2 Co‐Loading of p53 mRNA and CPX Results in Cell Killing In Vitro and Tumor Reduction In Vivo

**FIGURE 3:**
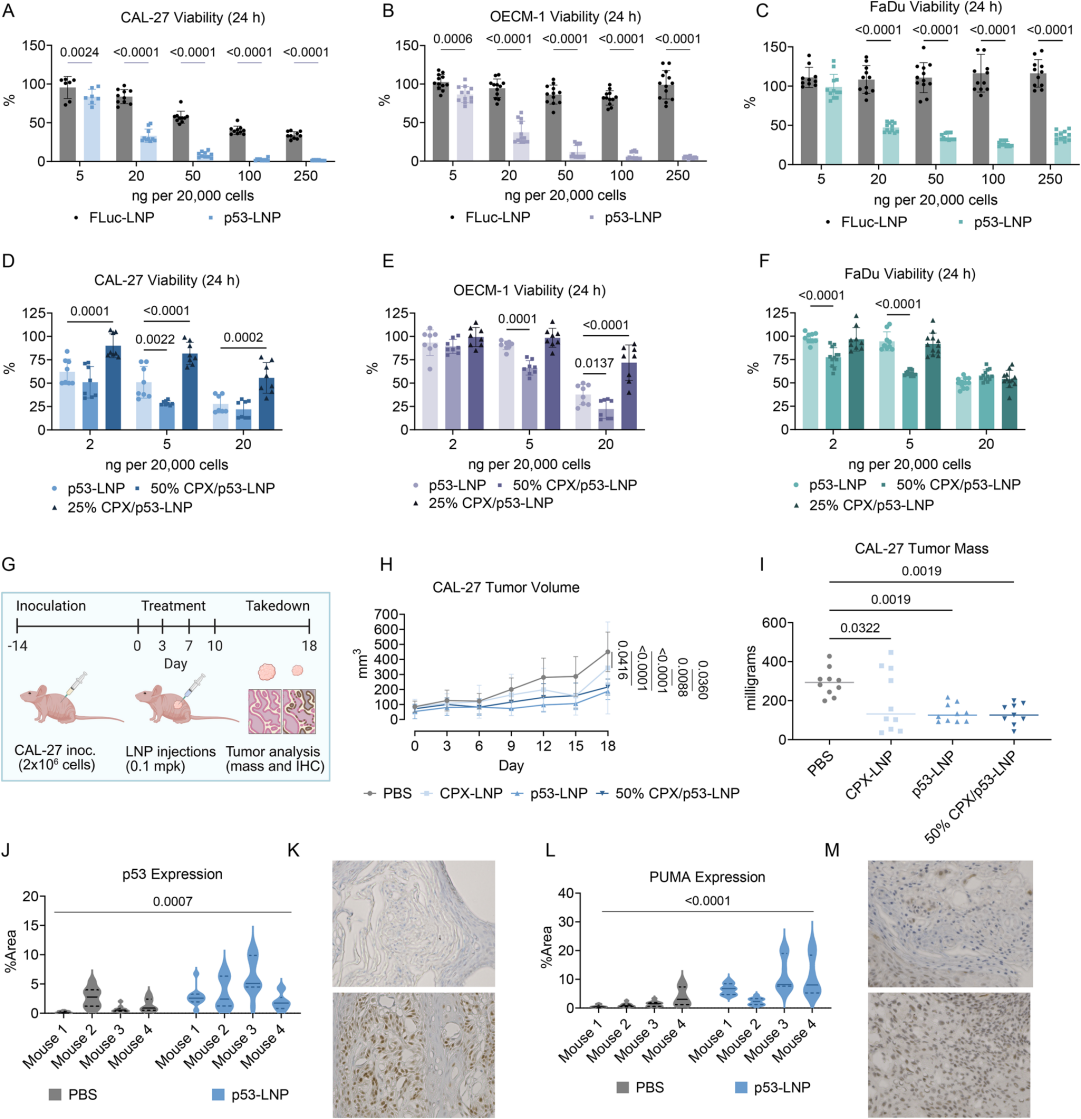
Cell killing properties of E10i‐494 p53 mRNA and CPX co‐therapy. (A–C), E10i‐494 was formulated with FLuc mRNA (FLuc‐LNP) and separately with human p53 mRNA (p53‐LNP). The two LNPs were incubated in (A) CAL‐27, (B) OECM‐1, and (C) FaDu cells at increasing dosages per 20 000 cells, and after 24 h, viability was measured. Viability is reported as mean ± SD of *n* = 8–12. (D–F), p53‐LNP, E10i‐494 reformulated with p53 mRNA and 50 mol% CPX (50% CPX/p53‐LNP), and E10i‐494 reformulated with 25 mol% CPX (25% CPX/p53‐LNP) were incubated in (D) CAL‐27, (E) OECM‐1, and (F) FaDu cells at 2, 5, and 20 ng of mRNA per 20 000 cells, and after 24 h, viability was measured. Viability is reported as mean ± SD of *n* = 8 for CAL‐27 and OECM‐1 cells and *n* = 9 for FaDu cells. (G), Nu/J mice were inoculated with two million CAL‐27 cells on each flank, and after 14 days, LNPs were administered intratumorally at an mRNA dose of 0.1 mpk four times over 10 days in each tumor. (H–M), Throughout the study, (H) tumor size was measured, and after 18 days, the mice were sacrificed, and (I) tumors were weighed. The PBS and p53‐LNP groups were further analyzed for protein expression, examining (J) p53 protein from the corresponding (K) IHC images as well as (L) PUMA protein from the corresponding (M) IHC images. Tumor volume and mass are reported as mean ± SD of *n* = 9 tumors from *n* = 5 mice. Protein expression is measured from *n* = 10 images, with a solid line representing the median and dashed lines representing quartiles. Representative IHC images are shown for each group. (A–C), Two‐tailed multiple unpaired *t*‐test with post hoc Holm–Šídák correction for multiple comparisons was used to compare FLuc‐LNP to p53‐LNP. (D–F, H, J, L), Two‐way ANOVA with post hoc Holm–Šídák correction for multiple comparisons was used to compare (D–F) p53‐LNP viability across LNP types for each dose, (H) tumor volume at each time point across groups, and (J) p53 and (L) PUMA %area between PBS and p53‐LNP groups. (I), One‐way ANOVA with post hoc Holm–Šídák correction for multiple comparisons was used to compare tumor mass to PBS.

After identifying E10i‐494 as the lead candidate, we reformulated the LNP with human p53 mRNA to assess whether the mRNA can induce OSCC killing in vitro. When comparing E10i‐494 formulated with FLuc mRNA (FLuc‐LNP) to the LNP formulated with human p53 mRNA (p53‐LNP), we found that the p53 mRNA induces dose‐dependent cellular toxicity in CAL‐27 (Figure 3A), OECM‐1 (Figure 3B), and FaDu (Figure 3C) cells. A further increase in p53‐induced toxicity was observed at 48 h (Figure S2A–C). Interestingly, FLuc‐LNP induced toxicity in CAL‐27 cells, while in OECM‐1 and FaDu cells, no FLuc‐LNP toxicity was observed. This is likely a result of the cellular differences among the OSCC lines, as observed with the mCherry results, where some cell lines may be more sensitive to LNP delivery. When comparing E10i‐494 encapsulating p53 mRNA against PTEN mRNA, another TSG commonly inactivated in OSCC, the p53‐LNP induced 2‐ to 4‐fold greater toxicity than with PTEN mRNA (PTEN‐LNP) in all three OSCC lines (Figure S3). With the efficacy of p53 mRNA demonstrated in vitro, we next evaluated CPX. Since CPX has not been tested in OSCC lines, we performed a dose response assay with free CPX and found that the free drug reduced viability to 50% at concentrations around 100 ng per 20 000 cells in all three cell lines (Figure S4A–C). As CPX has low water solubility and high solubility in ethanol, we formulated four LNPs encapsulating CPX at 2, 10, 25, and 50 mole percent (mol%) with FLuc mRNA by dissolving CPX in the ethanol phase along with the other lipid excipients.

The four CPX LNPs were then evaluated for their chemotherapeutic properties by incubating them in CAL‐27, OECM‐1, and FaDu cells at doses of 20 ng per 20 000 cells. While no decrease in viability was observed at 24 h, at 48 h, the 50 and 25 mol% CPX LNPs resulted in viabilities of approximately 75%, a modest but noticeable decrease (Figure S5A–C). To determine the combination effect of CPX and p53, we reformulated E10i‐494 with p53 mRNA containing either 25 mol% CPX (25% CPX/p53‐LNP) or 50 mol% CPX (50% CPX/p53‐LNP). Upon dosing in the three cell lines at 2, 5, and 20 ng per 20 000 cells, we observed improved cell killing in all three cell lines for the 50% CPX/p53‐LNP compared to the p53‐LNP, especially at the 5 ng dose, whereas 25% CPX/p53‐LNP facilitated similar or higher viability compared to p53‐LNP. The greatest disparities in viability were observed at the 5 ng dose. For CAL‐27 cells at a 5 ng dose, the addition of 50 mol% CPX resulted in a decrease from 50% to 30% viability compared to p53‐LNP, whereas for OECM‐1 and FaDu cells, the viability decreased from 100% to 65% (Figure 3D–F). With the 50% CPX/p53‐LNP inducing the lowest viability, it was identified as the lead candidate.

We then explored whether separately adding FLuc‐LNPs with 50% mol CPX (CPX‐LNP) and p53‐LNP would have the same combined impact as the co‐formulated 50% CPX/p53‐LNP. To explore this, we dosed CAL‐27 and FaDu cells at concentrations of 5, 20, and 50 ng per 20 000 cells with CPX‐LNP, p53‐LNP, 50% CPX/p53‐LNP, and co‐dosed p53‐LNP and CPX‐LNP (CPX‐LNP+p53‐LNP) and analyzed viability after 24 h. For the CPX‐LNP+p53‐LNP group, we dosed cells based on the encapsulated p53 concentration, meaning these groups received approximately twice the dose of LNP. At the lowest and highest doses, we found no difference between the 50% CPX/p53‐LNP and CPX‐LNP+p53‐LNP groups, whereas for the medium dose of 20 ng, the latter group induced a 10% reduction in viability compared to the 50% CPX/p53‐LNP group (Figure S6A–B). Although there may be potential benefit in simultaneously administering two separate formulations, it requires dosing twice as much LNP, which itself can induce non‐specific toxicity. To minimize potential off‐target toxicity, we proceeded with the co‐formulated platform.

To understand how CPX encapsulation impacts LNP endosomal escape, the LNPs were reformulated with 0%, 25%, or 50% CPX with Cy5‐tagged GFP mRNA and the lipophilic dye, DiR, and flow cytometry was performed after incubating the LNPs in CAL‐27 and FaDu cells. Here, Cy5 represents mRNA accumulation, GFP signifies mRNA translation, and DiR corresponds to LNP uptake. After dosing at either 10 or 100 ng per 200 000 cells, we found that at both the low and high doses, CAL‐27 and FaDu cells had near 100% Cy5‐mRNA and DiR accumulation (Figure S7A–H). However, GFP positivity ranged from 5% to 25% at the low dose, with the 25% and 50% CPX LNPs having worse GFP translation than the unencapsulated versions. At the higher dose, only marginal differences in GFP positivity were observed between the three LNPs; however, the 0% CPX LNP had more GFP expression per cell when examined by median fluorescent intensity (MFI). Overall, this indicates that CPX encapsulation does not reduce the ability of LNPs to enter oral cancer cells but does slightly hinder endosomal escape. This also may explain why LNPs with 50 mol% CPX improve cell killing over 25 mol% CPX, as the large increase in drug loading provides overall benefits over a minute diminishment in RNA endosomal escape.

With the chemotherapeutic properties of p53 mRNA and CPX demonstrated in vitro, we moved to a proof‐of‐concept in vivo study. Two million CAL‐27 cells were inoculated in Nu/J mice on each flank, and after 2 weeks, PBS, CPX‐LNP, p53‐LNP, and 50% CPX/p53‐LNP were intratumorally administered at 0.1 mg mpk four times over 10 days on each tumor (Figure 3G). Tumor volumes were measured throughout the course of the experiment, and after a total of 18 days from the first injection, the mice were sacrificed, the tumor mass was measured, and some of the tumors were processed using immunohistochemistry (IHC). While the CPX‐LNP induced a modest decrease in tumor volume and mass, the p53‐LNP and 50% CPX/p53‐LNP significantly delayed tumor growth by one‐third of the PBS burden. This demonstrates that for CAL‐27 xenograft tumors, p53 mRNA, and not CPX, is sufficient to diminish tumor burden (Figure 3H,I). Additionally, no weight loss was observed (Figure S8). IHC analysis of tumor tissues from the PBS control and p53‐LNP groups showed that the latter had increased expression of p53 and its downstream target, PUMA, demonstrating that pro‐apoptotic pathways were activated because of the therapy (Figure 3J–M).

### 2.3 CPX Is Encapsulated Heterogeneously and Forms a Complex Microstructure

**FIGURE 4:**
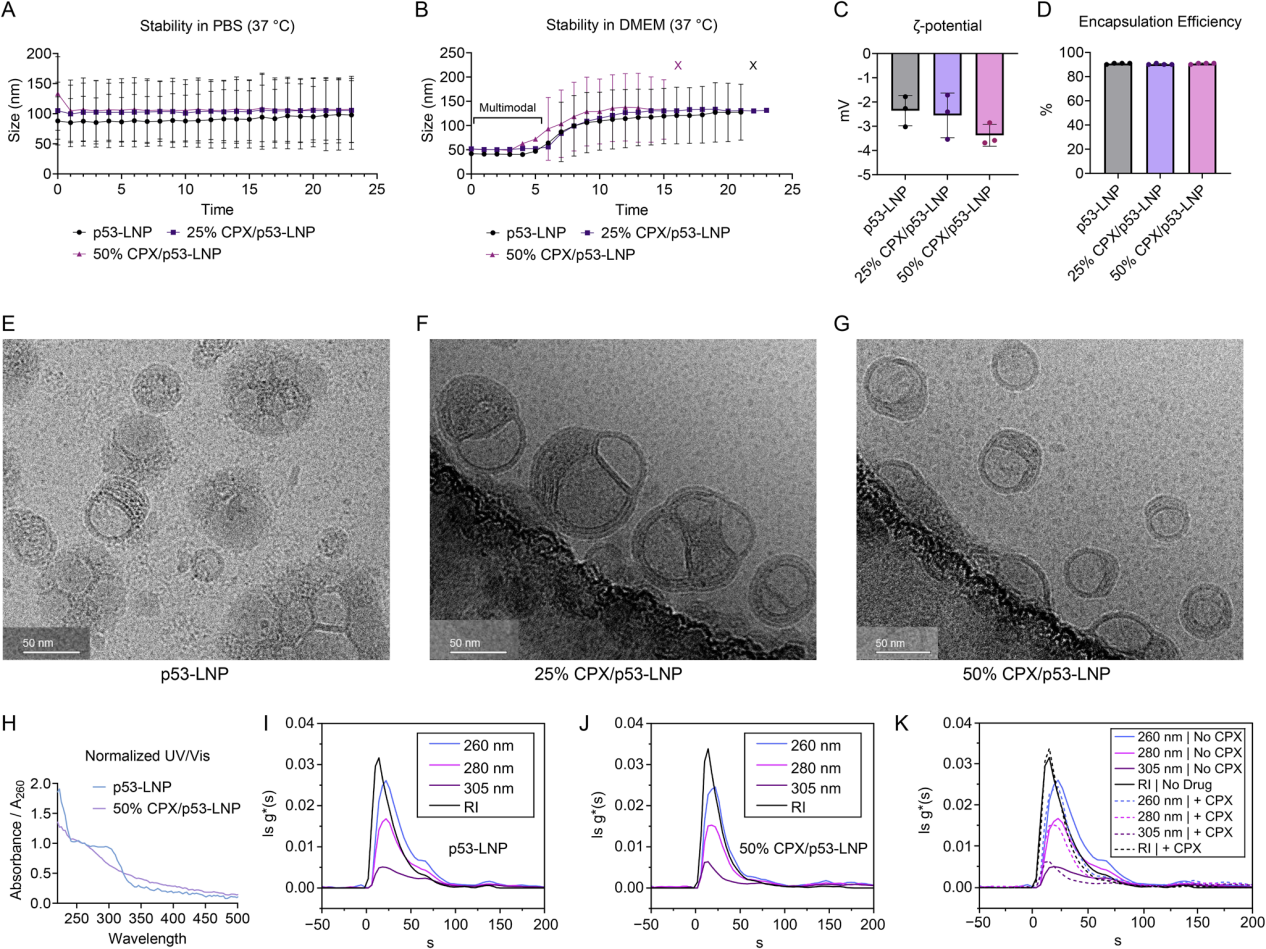
Physicochemical characterization of CPX‐containing LNPs. (A–B), Stability studies of E10i‐494 LNPs containing p53 mRNA (p53‐LNP), p53 mRNA and 25 mol% CPX (25% CPX/p53‐LNP), and p53 mRNA and 50 mol% CPX (50% CPX/p53‐LNP). LNP size was analyzed in (A) 1X PBS or (B) supplemented DMEM every hour for 24 h at 37°C via dynamic light scattering (DLS). Hydrodynamic diameter is reported as mean ± SD of *n* = 6; “x” represents the last measurement before a failed DLS measurement. (C), ζ‐potential of each LNP obtained via electrophoretic light scattering (ELS). (D), Relative p53 mRNA encapsulation efficiency determined by a RiboGreen Assay. (E–G), Cryogenic transmission electron microscopy (cryo‐TEM) images of (E) p53‐LNP, (F) 25% CPX/p53‐LNP, and (G) 50% CPX/p53‐LNP, with scale bars representing 50 nm. (H), UV/vis spectra of p53‐LNP and 50% CPX/p53‐LNP, normalized by the absorbance at 260 nm. (I–K),  ls–g*(*s*) distribution profiles from analytical ultracentrifugation (AUC) analyses of (I) p53‐LNP, (J) 50% CPX/p53‐LNP, and (K) overlayed LNPs using the program SEDFIT.

To further explore how CPX impacts LNP physicochemical features, we investigated different properties using an array of analytical tests. Dynamic light scattering was implemented to ascertain size, polydispersity index (PDI), and stability over time. The p53‐LNP, 25% CPX/p53‐LNP, and 50% CPX/p53‐LNP were diluted 10‐fold in either 1X PBS or supplemented Dulbecco's Modified Eagle Medium (DMEM), and hydrodynamic diameter and PDI were analyzed at 37°C every hour for 24 h. CPX encapsulation slightly increased particle size in PBS from 88 nm for p53‐LNP to 105 nm and 133 nm for 25% CPX/p53‐LNP and 50% CPX/p53‐LNP groups, respectively (Figure 4A). Over time, the p53‐LNP gradually increased in size to 98 nm, while the two CPX LNPs hovered around 106 nm. In DMEM, it took 6 h before stable particles were able to be observed, likely corresponding to protein corona formation (Figure 4B). All three particles gradually grew to approximately 130 nm, with 50% CPX/p53‐LNP becoming unstable and unable to be sized after 16 h, p53‐LNP becoming unstable after 22 h, and 25% CPX/p53‐LNP remaining stable during all 24 h of measurement. The ζ‐potential of the three LNPs was measured via electrophoretic light scattering and all formulations displayed a slightly negative surface charge of −3 mV, which is expected as CPX has a neutral charge, while the PEGylated lipid in all formulations has a negative charge (Figure 4C). CPX did not alter the relative encapsulation efficiency of p53 mRNA either, as determined by a RiboGreen Assay, with the three LNPs having 90% encapsulation (Figure 4D).

Utilizing cryogenic transmission electron microscopy (cryo‐TEM), we ascertained the morphology of the three LNPs. The p53‐LNP formulation appeared as a traditional Janus or bleb‐like architecture, with an electron‐dense and a less‐electron‐dense region (Figure 4E). The 25% CPX/p53‐LNP formulation was composed of a mixture of traditional bleb and di/tri‐bleb structures, the latter including two to three less‐electron‐dense pockets and a single electron‐dense region (Figure 4F). In comparison, the 50% CPX/p53‐LNP had a diverse mixture of blebs, di/tri‐blebs, and particles with multi‐blebs, consisting of four to eight less‐electron‐dense regions within a singular more electron‐dense region (Figure 4G), suggesting heterogeneous incorporation of CPX. We recently reported that LNPs have inherent subpopulations with unique sizes, shapes, and internal morphologies [29]. Thus, it is likely that CPX preferentially occupies certain subpopulations within the LNP, creating CPX‐concentrated LNPs. To further probe LNP heterogeneity, we performed sedimentation velocity analytical ultracentrifugation (SV‐AUC) on p53‐LNP and 50% CPX/p53‐LNP. By UV/vis spectroscopy, CPX‐LNP displays an enhanced optical signal at 305 nm, consistent with the reported UV/vis spectrum of free CPX (Figure 4H) [30]. Using SV‐AUC, we applied a least‐squares g*(s) model to identify both floating and sedimenting species and found that p53‐LNP and 50% CPX/p53‐LNP predominantly sediment, spanning *S* values of 0–100 (Figure 4I,J). p53‐LNP, which does not have CPX, has a broad signal at 305 nm corresponding to the scattering of photons by the LNPs, whereas the signal sharpens at a peak *S* value of 30 for 50% CPX/p53‐LNP. Overlaying the two plots, the 50% CPX/p53‐LNP has a higher 305 nm peak than the p53‐LNP, further confirming drug loading (Figure 4K). Consistent with the cryo‐TEM images, the 305 nm peak does not completely overlap with the LNP signal, represented by the absorbances at 260 nm and 280 nm, confirming that only certain populations have encapsulated CPX. The absence of a 305 nm signal near 0 S indicates the lack of free‐floating CPX, suggesting complete encapsulation.

### 2.4 p53 mRNA and CPX Activate Caspases in Cancer Cells and Repolarize Tumor‐Associated Macrophages

**FIGURE 5:**
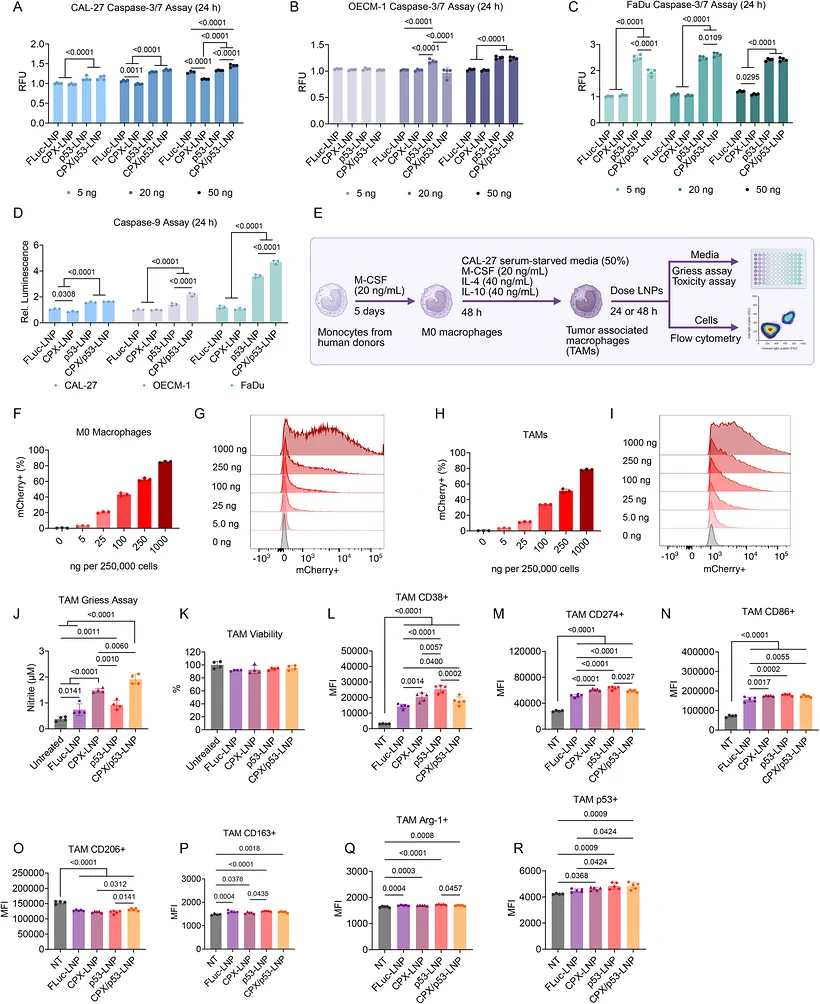
Mechanistic analysis of cell killing and macrophage polarization for the CPX/p53‐LNP platform. (A–C), E10i‐494 LNPs containing FLuc mRNA (FLuc‐LNP), 50 mol% CPX (CPX‐LNP), human p53 mRNA (p53‐LNP), and 50 mol% CPX and human p53 (CPX/p53‐LNP) were dosed in (A) CAL‐27, (B) OECM‐1, and (C) FaDu cells at 5, 20, and 50 ng per 20 000 cells. After 24 h, a fluorescence‐based caspase‐3/7 assay was performed on the cell supernatant, where fluorescence was calculated relative to untreated cells. Relative fluorescent units (RFU) are reported as mean ± SD of *n* = 4. (D), All four LNPs were incubated in CAL‐27, OECM‐1, and FaDu cells at a dose of 50 ng per 20 000 cells. After 24 h, a luminescence‐based caspase‐9 assay was performed on cell supernatant, where luminescence was calculated relative to untreated cells. Relative luminescence is reported as mean ± SD of *n* = 3. (E), Schematic of primary human monocyte polarization to M0 macrophages and TAMs using a combination of cytokines and serum‐depleted media from CAL‐27 cells. (F–I), E10i‐494 encapsulating mCherry mRNA was incubated with M0 macrophages and TAMs at increasing doses per 250 000 cells. After 24 h, flow cytometry was utilized to determine (F) mCherry+ M0 macrophages, (G) M0 macrophage fluorescence intensity, (H) mCherry+ TAMs, and (I) TAM fluorescence intensity. mCherry positivity is reported as mean ± SD of *n* = 3. Representative data are shown for fluorescence intensity plots. (J–K), All four LNPs were incubated in TAMs at a dose of 100 ng per 100 000 cells, and (J) after 24 h, a Griess assay was performed to measure nitrite production and (K) a viability assay was employed to determine TAM viability. (L–R), The four LNPs were dosed at 250 ng per 250 000 cells in TAMs and after 48 h, flow cytometry was used to examine (L) CD38, (M) CD274, (N) CD86, (O) CD206, (P) CD163, (Q) Arg‐1, and (R) p53 MFI. Nitric oxide concentration and cellular viability are reported as mean ± SD of *n* = 4, while MFI is reported as mean ± SD of *n* = 5. (A–D), Two‐way ANOVA with post hoc Holm–Šídák correction for multiple comparisons was used to compare (A–C) RFU across LNP types for each dose and (D) relative luminescence across LNP types for each cell line. (J–R), One‐way ANOVA with post hoc Holm–Šídák correction for multiple comparisons was used to compare (J) nitrite concentration, (K) cellular viability, and (L–R) MFI across each LNP type.

To probe the mechanism of tumor cell death and investigate whether the therapies can modulate the TME, we performed a series of mechanistic studies. First, we measured caspase activity in tumor cells. Caspase enzymes are cysteine proteases that facilitate apoptosis, which can be triggered extracellularly and intracellularly. Many chemotherapeutics, once delivered into the cell, mediate intracellular caspase‐based apoptosis, where mitochondria release cytochrome C that induces the formation of an apoptosome, a multimeric protein complex that recruits and activates caspase‐9, an initiator caspase [31]. Once caspase‐9 is engaged, a caspase activation cascade occurs, leading to the activation of executioner caspases, including caspase‐3 and caspase‐7, and consequently, cell death. To explore the combinatory effect of p53 and CPX on caspase activity, FLuc‐LNP, p53‐LNP, CPX‐LNP, and 50% CPX/p53‐LNP (now referred to as CPX/p53‐LNP) were dosed in CAL‐27, OECM‐1, and FaDu cells at 5, 20, and 50 ng per 20 000 cells. After 24 h, a fluorescence‐based caspase‐3/7 assay was conducted, which measures caspase‐3/7 activity. Normalizing to untreated wells, we found that in CAL‐27 cells, p53‐LNP increases caspase‐3/7 activity in each dose, but for the 50 ng dose, the CPX/p53‐LNP group had 10% higher fluorescence than p53‐LNP (Figure 5A), indicating CPX increases caspase‐3/7 activity in CAL‐27 cells. In contrast, CPX did not enhance caspase‐3/7 activity in OECM‐1 and FaDu cells, as the p53‐LNP and CPX/p53‐LNP groups maintained the same fluorescence at all doses (Figure 5B,C). Out of the three cell lines, FaDu cells produced the most caspase‐3/7 upon LNP treatment, as the p53‐LNP and CPX/p53‐LNP groups produced 2.5‐fold higher fluorescence than baseline, compared to 1.5‐fold and 1.25‐fold in CAL‐27 and OECM‐1 cells, respectively. We then examined caspase‐9 activity utilizing a luminescence‐based reporter system. As the 50 ng dose induced the strongest caspase‐3/7 activity, we dosed the three cell lines for the caspase‐9 studies at 50 ng. After 24 h, we observed an opposite trend from the previous study. Here, CPX/p53‐LNP enhanced caspase‐9 activity by 2‐fold and 1.5‐fold compared to p53‐LNP in OECM‐1 and FaDu cells, respectively, whereas there were no differences in activity for the two LNPs in CAL‐27 cells (Figure 5D). Similar to the caspase‐3/7 assays, treatment in FaDu cells induced the highest expression of caspase‐9 compared to the other cell lines. Thus, CPX enhances p53‐mediated caspase activity, but the specific caspase depends on the cell line. When examining other potentially related pathways, we found that none of the LNPs induced meaningful increases in mitochondrial permeability (Figure S9) nor reactive oxygen species (Figure S10).

To investigate the effect of the therapies on the TME, we examined macrophage polarization, focusing on TAMs as the dominant innate immune cell in OSCC tumors, which are associated with poorer outcomes [32]. We first obtained monocytes from healthy human donors and incubated them with macrophage colony‐stimulating factor (M‐CSF) for 5 days to generate M0 macrophages (Figure 5E). To avoid unintentional polarization, we cultured the cells in human serum, rather than bovine serum. After converting to M0 macrophages, we further cultured the cells with IL‐4, IL‐10, and conditioned medium from serum‐starved CAL‐27 cells for 48 h to induce OSCC TAMs. To determine whether the E10i‐494 LNP, which was optimized for OSCC lines, can transfect macrophages, we reformulated the LNP with mCherry mRNA and performed a dose escalation study in both M0 macrophages and TAMs. Although more difficult to transfect, mCherry expression was observed in a dose‐dependent manner, where 80% of M0 macrophages and 75% of TAMs were mCherry positive at a dose of 1 000 ng per 250 000 cells after 24 h (Figure 5F–I). We then determined if the LNPs can promote nitric oxide formation, which is a pro‐inflammatory molecule characteristic of M1‐like macrophages, but is produced in minute concentrations in the TME [33]. TAMs were dosed at 100 ng per 100 000 cells with FLuc‐LNP, CPX‐LNP, p53‐LNP, and CPX/p53‐LNP for 24 h. Afterward, we performed a Griess assay, which examines nitrite, as nitric oxide is rapidly converted to nitrite in cell media. Using nitrite concentration as a proxy for nitric oxide, we observed that CPX‐LNP and CPX/p53‐LNP increased nitric oxide production in TAMs by 1.5‐fold and 2‐fold, respectively, compared to p53‐LNP, and 3‐fold and 4‐fold compared to FLuc‐LNP (Figure 5J). This suggests that CPX is the major driver of nitric oxide production, although p53, when combined with CPX, can further increase production. In addition, no obvious changes in cell viability were observed after dosing in TAMs, suggesting that CPX and p53 mRNA had no effects on the cell viability of macrophages (Figure 5K). A slight increase in nitric oxide production was observed in FLuc‐LNP compared to untreated cells, demonstrating that the innate inflammatory nature of LNPs can also induce nitric oxide formation.

To probe how the LNPs modulate TAM phenotype, we repeated the treatment regimen and examined the cells by flow cytometry after 48 h. We found that both the LNP itself and the encapsulated p53 mRNA contribute separately to repolarizing TAMs to a more M1‐like phenotype. The MFI of M1‐like markers such as CD38 and CD274 was increased by CPX‐LNP, p53‐LNP, and CPX/p53‐LNP, but most strongly by p53‐LNP (Figure 5L,M). The M1‐like marker CD86 was elevated for all groups, including FLuc‐LNP, indicating that the inflammatory nature of the LNP was the driving factor for CD86 upregulation (Figure 5N). The MFI of the M2‐like marker CD206 was also lowered by all LNPs, whereas for CD163 and arginase‐1 (Arg‐1), only minor differences in MFI were observed (Figure 5O–Q). Lastly, both p53‐LNP and CPX/p53‐LNP facilitated greater p53 MFI in TAMs, due to the delivery of p53 mRNA, while CPX‐LNP slightly increased p53 MFI (Figure 5R). The flow results and Griess assay describe a complex interaction between the LNP, CPX, and p53 mRNA, where each is contributing to the repolarization of TAMs to a more M1‐like phenotype. Although further studies are needed to better define the mechanism, these preliminary results suggest that CPX can increase the production of pro‐inflammatory nitric oxide, while p53 mRNA and the LNP vehicle itself alter TAM phenotypes.

### 2.5 CPX Is the Major Driver of Tumor Reduction in p53‐Resistant MOC‐2 Syngeneic Tumors

**FIGURE 6:**
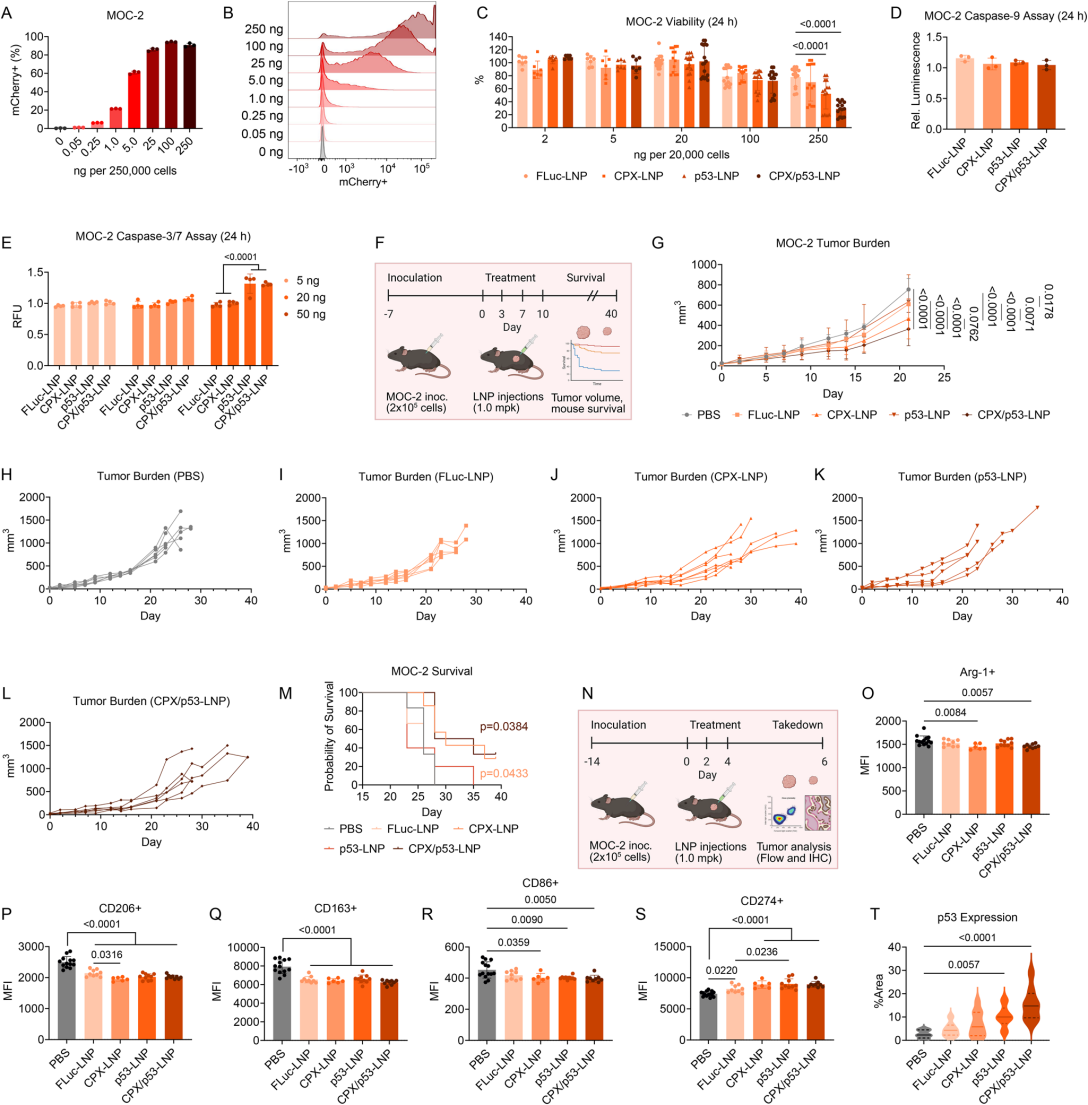
Evaluation of LNP co‐therapy in a syngeneic murine tumor model. (A, B), E10i‐494 encapsulating mCherry mRNA was incubated with MOC‐2 cells at increasing doses per 250 000 cells. After 24 h, flow cytometry was utilized to determine (A) mCherry+ MOC‐2 cells and (B) MOC‐2 fluorescence intensity. mCherry positivity is reported as mean ± SD of *n* = 3. Representative data are used for the fluorescence intensity plots. (C), E10i‐494 LNPs containing FLuc mRNA (FLuc‐LNP), 50 mol% CPX and FLuc mRNA (CPX‐LNP), mouse p53 mRNA (p53‐LNP), and 50 mol% CPX and mouse p53 (CPX/p53‐LNP) were incubated in MOC‐2 cells at increasing doses per 20 000 cells, and after 24 h, viability was measured. Cellular viability is reported as mean ± SD of *n* = 8 for 2 ng and 5 ng doses and *n* = 12 for all other doses. (D), All four LNPs were incubated in MOC‐2 cells at a dose of 50 ng per 20 000 cells. After 24 h, a luminescence‐based caspase‐9 assay was performed on cell supernatant, where luminescence was calculated relative to untreated cells. Relative luminescence is reported as mean ± SD of *n* = 3. (E), All four LNPs were incubated in MOC‐2 cells at doses of 5, 20, and 50 ng per 20 000 cells. After 24 h, a fluorescence‐based caspase‐3/7 assay was performed on the cell supernatant, where fluorescence was calculated relative to untreated cells. Relative fluorescent units (RFU) are reported as mean ± SD of *n* = 4. (F), C57BL/6J mice were inoculated with 200 000 MOC‐2 cells on the right flank, and after 7 days, LNPs were administered intratumorally at an mRNA dose of 1.0 mpk four times over 10 days. (G–L), 7 days after the initial inoculation, tumor volumes were measured (G) comparatively for up to 22 days as well as individually for the (H) PBS, (I) FLuc‐LNP, (J) CPX‐LNP, (K) p53‐LNP, and (L) CPX/p53‐LNP groups. Tumor burden is reported as mean ± SD of *n* = 6 for PBS and *n* = 5 for all other groups. (M), Kaplan‐Meier survival curves for all five groups. (N), C57BL/6J mice were inoculated with 200 000 MOC‐2 cells on the right flank, and after 14 days, LNPs were administered intratumorally at an mRNA dose of 1.0 mpk three times over 4 days. (O–S), After 20 days from the initial inoculation, the mice were sacrificed, and the tumors were harvested and analyzed by flow cytometry to examine (O) Arg‐1, (P) CD206, (Q) CD163, (R) CD86, and (S) CD274. MFI is reported as mean ± SD of *n* = 14 for PBS, *n* = 10 for p53‐LNP, *n* = 9 for FLuc‐LNP and CPX/p53‐LNP, and *n* = 6 for CPX‐LNP. (T), Additionally, tumors were characterized by IHC for p53 expression. Protein expression is measured from *n* = 8 images with a solid line representing the median and dashed lines representing quartiles. (C, E, G), Two‐way ANOVA with post hoc Holm–Šídák correction for multiple comparisons was used to compare (C) cellular viability across LNP types for each dose, (E) RFU activity across LNP types for each dose, and (G) tumor volume across LNP types for the time point. (D, O–T), One‐way ANOVA with post hoc Holm–Šídák correction for multiple comparisons was used to compare (D) relative luminescence, (O–S) MFI, and (T) %area across each LNP type. (M), Kaplan‐Meier estimator with post hoc Holm–Šídák correction for multiple comparisons was used to compare survival curves to PBS.

Having demonstrated the potency of the platform in human OSCC, we then evaluated whether this co‐therapy approach could improve outcomes in a syngeneic murine tumor model formed by MOC‐2 cells, an aggressive mouse OSCC line. We first reformulated E10i‐494 with mCherry mRNA and incubated the LNP in MOC‐2 cells at increasing doses to evaluate the transfection efficiency. We found that at 25 ng per 250 000 cells, E10i‐494 transfected almost 80% of MOC‐2 cells, which is on par with the human OSCC lines (Figure 6A,B). However, when assessing the potency of CPX‐ and p53‐loaded LNPs in MOC‐2 cells, the latter using a mouse p53 template, after 24 h, 250 ng was required to elicit cell death – a 10‐fold greater dose than what was needed for the human OSCC lines (Figure 6C). After 48 h, there were further increases in toxicity at the 250 ng dose, with some increase at the 100 ng dose (Figure S11). No significant changes in caspase‐9 production were observed at a dose of 50 ng per 20 000 cells for all LNPs (Figure 6D), but an increase in caspase‐3/7 activity was observed upon treatment with the highest dose of p53‐LNP and CPX/p53‐LNP, indicating functional murine p53 mRNA (Figure 6E). We then examined whether our therapeutic system could extend survival in C57BL/6J mice inoculated with MOC‐2 cells. LNPs were administered at a dose of 1.0 mpk four times over 10 days, and mice were monitored for up to 40 days from the initial LNP injection (Figure 6F). No loss in mouse weight was observed during the LNP administration period (Figure S12). After 22 days from the first injection, the CPX‐LNP and CPX/p53‐LNP groups had lower tumor volumes than the PBS, FLuc‐LNP, and p53‐LNP groups, with the co‐therapy inducing the lowest tumor volume (Figure 6G). The CPX‐LNP and CPX/p53‐LNP groups also maintained a flatter tumor growth curve during the 40‐day experiment compared to the other groups (Figure 6H–L). p53‐LNP only slightly prolonged survival, but CPX‐LNP and CPX/p53‐LNP increased survival by over a week, suggesting that CPX can enhance the anti‐tumor effect and prolong survival in mice burdened with tumors that respond poorly to p53 therapy (Figure 6M).

To determine the mechanism underlying the reduction in tumor burden in this syngeneic model, we subcutaneously inoculated MOC‐2 cells into new C57BL/6J mice (Figure 6N). The four LNPs were injected intratumorally into mice bearing MOC‐2 tumors three times every 2 days at a dose of 1.0 mpk and then dissected 48 h after the final dose. Serum was isolated to examine systemic toxicity markers, and tumors were dissected for analysis by flow cytometry and IHC. In alignment with the negligible decrease in weight loss, all treated mice had similar levels of aspartate aminotransferase (AST), alanine aminotransferase (ALT), urea, and creatinine compared to mice treated with PBS (Figure S13A–D). Additionally, flow cytometry on macrophage markers indicated a general decrease in MFI of M2‐like markers, including Arg‐1, CD206, and CD163, with the CPX‐LNP group generally having the lowest values among all groups (Figure 6O–Q). Meanwhile, for M1‐like markers, a more complex picture was observed. There were slight decreases in CD86 MFI for the CPX‐LNP, p53‐LNP, and CPX/p53‐LNP groups, while those same groups also had greater increases in CD274 MFI compared to the PBS group (Figure 6R,S). Moreover, none of the LNP groups induced greater T cell infiltration, suggesting that the primary therapeutic mechanism is driven by macrophages, rather than T cells (Figure S14A–B). IHC of p53 protein production indicated increased p53 expression for both the CPX‐LNP and p53‐LNP groups and substantial p53 signal for the CPX/p53‐LNP group, suggesting that CPX aids in p53 production (Figure 6T).

## 3 Discussion

OSCC is a debilitating disease due to the importance and multifunctionality of the oral cavity. Mainline treatments, including surgery and chemoradiation, can halt disease progression but come at the cost of further damage to the oral cavity, where in most cases, the tongue is either partially or completely resected. Many patients have also not benefited from current state‐of‐the‐art immunotherapies, including cetuximab and checkpoint blockade inhibitors due to a lack of response. While other cellular and immunotherapies have facilitated remission for different cancers, OSCC has largely been left out of testing. Thus, we aimed to develop a co‐therapy platform based on the biology of OSCC development, by targeting both the inactivation of TSGs and the immunosuppressive TME. A hallmark of this disease is that its progression relies more on the inactivation and mutation of TSGs, rather than the overexpression of multiple oncogenes. This allows it to be well‐suited for mRNA‐based approaches that can facilitate the expression and functional activation of TSGs in OSCC.

LNPs are the primary vehicle for mRNA delivery in the clinic; however, the complex structure of LNPs also allows for encapsulation of therapeutics other than nucleic acids [34]. For hydrophilic compounds, intermolecular interactions, such as electrostatic interactions, with the LNP components must be leveraged to achieve successful encapsulation, as otherwise, the compound will be mainly located outside of the LNP in the surrounding buffer because of favorable entropic and enthalpic conditions. Similarly, therapeutics that are too hydrophobic may not dissolve in ethanol and thus are unable to be incorporated into the LNP. CPX is uniquely situated to be encapsulated due to its combination of low water solubility and high solubility in ethanol, allowing it to be co‐dissolved in the ethanolic phase during LNP formulation. There are likely many other drug candidates that have favorable solubility properties and can also be formulated with nucleic acids for potent co‐therapy. This pool of small‐molecule drugs can be expanded by altering the aqueous and organic phases during LNP formulation.

While prior efforts to apply LNPs in HNSCC have focused on optimizing formulation parameters around pre‐existing lipids such as 7C1 without evaluating ionizable lipid chemistry itself, the present study is, to our knowledge, the first to systematically screen structurally distinct ionizable lipids for nucleic acid delivery for OSCC. The superior performance of E10i‐494 over FDA‐approved SM‐102 and ALC‐0315, lipids originally optimized for intramuscular vaccine delivery, as well as over the liver‐tropic C12‐200 underscores that ionizable lipid design must be tailored to the target tissue and that established lipids cannot be assumed to generalize across disease contexts.

Our approach not only investigated the delivery of two therapeutics but also examined the role of simultaneous uptake in cancer cells and macrophages. Many cancer cells readily take up LNPs due to their high metabolism and dependence on cholesterol for membrane biogenesis; however, macrophages are also a repository for LNPs due to their phagocytic nature [35]. As TAMs are one of the dominant immune cells in the oral TME, devising nanoparticles to both kill cancer cells and reshape the TME would greatly increase the potency of the drug delivery platform. While p53 has been demonstrated to repolarize M2‐like macrophages to M1‐like macrophages, our results suggest, that p53 can repolarize TAMs within the oral TME of OSCC [18]. This is significant since previous p53‐based therapies have focused solely on targeting cancer cells, meaning that possible benefits from targeting immune cells have been overlooked. Similar to the growing evidence that traditional chemotherapeutics, including doxorubicin, activate the immune system, we demonstrate that p53 likewise possesses a similar chemoimmunotherapeutic potential, although more studies are needed to further elucidate this mechanism [36]. Second, our results demonstrated that CPX can polarize TAMs into M1‐like phenotypes characteristic of increased nitric oxide production. Even though CPX has shown immunomodulatory effects in certain settings, including boosting T cell infiltration in a syngeneic mouse 4T1 breast tumor model [37] and suppressing osteoclast formation in an ovariectomy‐induced osteoporosis model in mice [38], little is known about its role in macrophage polarization. A recent study showed that blocking spermidine‐mediated hypusination of the eukaryotic translation initiation factor 5A (eIF5A) inhibited OSCC‐induced M2‐like TAMs [39]. Several studies demonstrate that CPX exerts its anti‐tumor effects by inhibiting the activity of deoxyhypusine hydroxylase (DOHH), one of the key enzymes responsible for eIF5A hypusination [40, 41]. Further studies are warranted to explore whether CPX skews OSCC‐induced M2‐like TAMs into an M1‐like phenotype by blocking DOHH‐mediated eIF5A hypusination.

Although the cell‐killing potential of p53 is well‐established, we found that CPX alone encapsulated in an LNP is a poor chemotherapeutic, yet in combination with p53 mRNA, CPX enhances caspase activity. More interestingly, the specific caspase affected by CPX depended on the OSCC line, where the tested cell lines were derived from different anatomical regions of the oral cavity, suggesting that each OSCC line has a unique apoptosis pathway due to its intrinsic heterogeneity. These results emphasize the need for testing multiple heterogeneous cell lines for OSCC, as well as other types of cancers, to properly gauge how the therapeutic platform will respond to phenotype differences. This is particularly important for translating a product to human OSCC, which is highly heterogeneous. The patient‐to‐patient variability is one reason that previous p53‐based therapies have failed in the clinic. Although OSCC tumors are broadly classified as p53‐deficient, individual cells within a tumor are heterogeneous: some retain wild‐type p53, while others harbor gain‐of‐function mutations that can dominantly suppress wild‐type p53 activity [42]. To account for this, we tested our platform in MOC‐2 cells, a murine OSCC line, which we found to be resistant to mouse p53 mRNA. Despite this tolerance, the addition of CPX was able to reduce tumor burden and extend survival in a MOC‐2 syngeneic mouse model due to its chemotherapeutic and immunomodulatory behavior. Thus, this platform has the potential to treat OSCC broadly, even in cases where one of the therapies is ineffective.

In summary, we have identified an optimal LNP for transfection in OSCC through a multi‐tiered approach, screening ionizable lipids with unique architecture for potent transfection and low liver delivery. We then leveraged the hydrophilic and hydrophobic domains in LNPs to co‐deliver p53 mRNA and the small molecule CPX. Rather than acting through a single mechanism, the therapies display context‐dependent combination effects: p53‐driven cell killing dominates in p53‐susceptible models, while CPX‐driven killing and macrophage repolarization dominate in p53‐resistant models. As a result, the co‐formulated platform reduces tumor burden across both contexts, offering a single delivery vehicle with breadth that neither monotherapy provides alone.

## 4 Methods

All animal use was in accordance with the guidelines and approval from the University of Pennsylvania's Institutional Animal Care and Use Committee (IACUC; protocols 806540 and 804466).

### 4.1 Materials

All non‐synthesized lipid excipients were purchased from Avanti Polar Lipids (Alabaster, AL, USA). Firefly luciferase mRNA with 5‐methoxyuridine substitutions and mCherry mRNA with N1‐methylpseudouridine substitutions were purchased from TriLink Biotechnologies (San Diego, CA, USA). Cy5‐tagged green fluorescent protein (GFP) mRNA with 5‐methoxyuridine substitutions was purchased from APExBIO (Houston, TX, USA). 1,2‐epoxyoctane, 1,2‐epoxydecane, 1,2‐epoxydodecane, and 1,2‐epoxytetradecane were purchased from TCI (Montgomeryville, PA, USA); Triton X‐100 was purchased from Alfa Aesar (Haverhill, MA, USA). 2‐{2‐[4‐(2‐{[2‐(2‐aminoethoxy)ethyl]amino}ethyl)piperazin‐1‐yl]ethoxy}ethan‐1‐amine (494) was purchased from Enamine (Kyiv, Ukraine). Ciclopirox was purchased from Santa Cruz Biotechnology (Dallas, TX, USA) and all other chemical reagents were purchased from MilliporeSigma (St. Louis, MO, USA).

### 4.2 Synthesis

Flash chromatography was performed on a Teledyne ISCO (Lincoln, NE, USA) CombiFlash NextGen 300+ equipped with evaporative light scattering detection (ELSD) using RediSep Gold silica gel disposable flash columns (Teledyne ISCO). Solvent evaporation was performed using a Büchi (New Castle, DE, USA) Rotavapor R‐300 System Professional. ^1^H and ^13^C NMR spectra were acquired in chloroform‐d using an Avance Neo 400 MHz spectrometer (Bruker, Billerica, MA, USA). NMR spectra were analyzed on MestReNova 14.3.0 (Mestrelab, Santiago de Compostela, Spain). Nominal mass accuracy LC‐MS data were obtained by use of a Waters (Milford, MA, USA) Acquity UPLC system equipped with a Waters TUV detector (254 nm) and a Waters SQD single quadrupole mass analyzer with electrospray ionization. Samples were prepared in 200 proof ethanol and injected onto an Acquity UPLC BEH C8 1.7 µm, 2.1 × 50 mm column with a 2 min wash followed by a gradient mobile phase from 50% water (1% trifluoroacetic acid) and 50% acetonitrile (1% trifluoroacetic acid) to 100% acetonitrile (1% trifluoroacetic acid) over 8 min. LC‐MS spectra were analyzed on MestReNova 14.3.0.

To a 3 mL vial (Thermo Fisher Scientific) was added the 494 polyamine core (1.0 equiv.), the corresponding epoxide (7.0 equiv.), and ethanol (0.3 mL). The reaction was stirred at 80°C for 48 h. Afterward, the crude product was diluted with dichloromethane (0.7 m) and purified via flash chromatography assisted by CombiFlash using a liquid injection into a 4 g RediSep Gold silica disposable flash column (Teledyne Isco). The mobile phase consisted of a gradient of 95% dichloromethane and 5% Ultra solution (75% dichloromethane, 22% methanol, and 3% aqueous ammonium hydroxide) to 80% dichloromethane and 20% Ultra solution over 35 min using a flow rate of 7 mL/min. The products were isolated as yellow to clear viscous oils. See Padilla et al. [26] for the full synthetic pathway, specific conditions, and spectra.

### 4.3 mRNA Production

Codon‐optimized human p53, human PTEN, and mouse p53 sequences were cloned into a plasmid with optimized 3′ and 5′ untranslated regions (UTRs) and containing a 101 polyA tail. mRNA was produced via in vitro transcription of the plasmid using 1‐methylpseudouridine‐modified nucleosides and co‐transcriptionally capped with CleanCap technology (TriLink). The mRNA was purified with cellulose to remove double‐stranded RNAs, and purified mRNAs were precipitated in ethanol and were re‐suspended in nuclease‐free water. Electrophoresis, dot blot, and endotoxin assays were performed for quality control. All mRNAs were stored in a −80°C freezer until use.

### 4.4 Lipid Nanoparticle Formulation

Cholesterol, 18:1 Δ9‐cis phosphoethanolamine (DOPE), 14:0 PEG2000 phosphoethanolamine (C14‐PEG_2000_), and an ionizable lipid were dissolved in ethanol at a molar ratio of 46.5:16:2.5:35, respectively. CPX was added to the ethanol phase for the LNPs containing CPX. The SM‐102 formulation was prepared using cholesterol, 1,2‐distearoyl‐sn‐glycero‐3‐phosphocholine (DSPC), and 1,2‐dimyristoyl‐rac‐glycero‐3‐methoxypolyethylene glycol‐2000 (DMG‐PEG_2000_), and the SM‐102 ionizable lipid at a molar ratio of 38.5:10:1.5:50, respectively. The ALC‐0315 formulation was prepared by combining cholesterol, DSPC, ALC‐0159, and the ALC‐0315 ionizable lipid at a molar ratio of 42.7:9.4:1.6:46.3, respectively. The aqueous phase was prepared by dissolving the corresponding mRNA in 10 mm citrate buffer at pH 3 (Teknova, Hollister, CA, USA). The volume ratio of the ethanol phase to aqueous phase was 1:3 with a 10:1 weight ratio of the corresponding ionizable lipid and mRNA. The citrate and ethanol phases were loaded into glass syringes (Hamilton Company, Reno, NV, USA) and connected to a Pump 33 DDS syringe pump (Harvard Apparatus, Holliston, MA, USA) attached to a microfluidic device with a staggered herringbone micromixer design. Microfluidic devices were fabricated in polydimethylsiloxane utilizing standard soft lithographic procedures [27]. A two‐step exposure process was used to create the SU‐8 master with positive channel features on a silicon wafer, where each mixing channel is 4 cm in length. The syringes were injected into the microfluidic device using flow rates of 1.8 and 0.6 mL/min for the aqueous and ethanol phases, respectively.

After formulation, LNPs were dialyzed against 1X PBS at pH 7.4 in 0.5 or 3 mL 20 kDa molecular weight cutoff (MWCO) cassettes (Thermo Fisher Scientific) for at least 2 h at room temperature. After dialysis, the LNPs were passed through a 0.22 µm syringe filter (Genesee Scientific, El Cajon, CA, USA) and stored at 4°C until further use. When necessary, LNPs were concentrated using Amicon 50k regenerated cellulose centrifugal filters (Millipore Sigma) pre‐rinsed with 1X PBS, centrifuging at 700 g until the desired volume was reached.

### 4.5 Encapsulation Efficiency

Relative encapsulation efficiency and encapsulated mRNA concentration were measured by a Quant‐iT RiboGreen assay (Thermo Fisher Scientific). Each sample was diluted 100‐fold in either 1X tris‐EDTA (TE) buffer or 1X TE buffer containing 1% (v/v) Triton X‐100. The Triton X‐100 samples were mixed thoroughly and allowed to incubate for 5 min to achieve complete lysis of the samples. A standard curve was generated by diluting the corresponding mRNA used during formulation to concentrations ranging from 2.00 to 31.3 ng/mL in 1X TE buffer. LNPs in TE buffer and LNPs in Triton X‐100 were plated in quadruplicate, while the mRNA standards were plated in duplicate in black 96‐well plates (Greiner, Kremsmünster, Austria). Afterward, the RiboGreen fluorescent detection reagent was added per the manufacturer's instructions. The plate was then wrapped in aluminum foil and shaken on a plate shaker at 200 rpm for 5 min. Afterward, fluorescence intensity was analyzed on an Infinite 200 Pro plate reader (Tecan, Morrisville, NC, USA) using the Tecan i‐control 3.9.1.0 software at an excitation wavelength of 485 nm and an emission wavelength of 525 nm. RNA concentration was determined via a standard curve estimated from a univariate least squares linear regression (LSLR). Relative encapsulation efficiency was calculated as $\mathrm{EE}\;=\frac{B-A}{B}\;*100\%$, where A is the measured unencapsulated mRNA in TE buffer, and B is the measured mRNA content after LNP lysis in Triton X‐100. The encapsulated mRNA concentration was calculated by [conc] =  B − A. Encapsulation efficiencies are reported as mean ± standard deviation.

### 4.6 In Vitro Culturing

CAL‐27 (CRL‐2095) and FaDu (HTB‐43) lines were purchased from ATCC (Manassas, VA, USA), MOC‐2 (EWL002‐FP) from Kerafast (Newark, CA, USA), and OECM‐1 (SCC180) lines were purchased from MilliporeSigma. CAL‐27, OECM‐1, and FaDu cells were cultured in tissue‐treated T75 flasks (Thermo Fisher Scientific) with Dulbecco's modified Eagle medium with *L*‐glutamine (DMEM; Gibco, Dublin, Ireland) supplemented with 10% fetal bovine serum (FBS; Gibco), and 1% penicillin‐streptomycin (P/S; Gibco). MOC‐2 cells were cultured in tissue‐treated T75 flasks in 2:1 IMDM (Thermo Fisher Scientific) and Hams Nutrient Mixture (Thermo Fisher Scientific) supplemented with 10% FBS, 1% P/S, 5 mg/L insulin (MilliporeSigma), 40 µg/L hydrocortisone (MilliporeSigma), and 5 µg/L epidermal growth factor (EGF; MilliporeSigma).

Primary human monocytes were obtained from healthy donors via the Perelman School of Medicine Human Immunology Core. The cells were incubated in non‐tissue‐treated T75 flasks containing Roswell Park Memorial Institute (RPMI) 1640 media (Gibco) supplemented with 10% human serum (MilliporeSigma), 1% P/S, and 20 ng/mL recombinant human macrophage colony‐stimulating factor (M‐CSF; PeproTech, Cranbury, NJ, USA) for 5 days at a maximum cell density of 1 million cells per mL to obtain M0 macrophages. The media was replaced every 2 or 3 days. To polarize M0 macrophages to TAMs, the M0 macrophages were cultured in 1:1 CAL‐27 tumor conditioned media and RPMI supplemented with 10% human, 1% P/S, 20 ng/mL M‐CSF, 40 ng/mL human recombinant IL‐4 (PeproTech), and 40 ng/mL human recombinant IL‐10 (PeproTech) for 48 h. CAL‐27 tumor conditioned media was generated by plating 10 million CAL‐27 cells in a tissue‐treated T175 flask (Thermo Fisher Scientific) with 100 mL of supplemented DMEM. After 24 h, the media was removed, the cells were washed with sterile 1X PBS, and 50 mL of DMEM containing 1% P/S and no FBS was added. After an additional 48 h, the media were transferred to a centrifugal tube, centrifuged at 300 g for 5 min, and the supernatant was collected as the tumor conditioned media.

All cells were cultured at 37°C in a humidified incubator with 5% CO_2_ and routinely tested for mycoplasma contamination through the University of Pennsylvania's Cell Center Stockroom. Cells were counted using a Countess 3 automated cell counter (Thermo Fisher Scientific) using 1:1 cell solution and Trypan Blue Stain (Thermo Fisher Scientific) for cancer cells and no Trypan Blue Stain for monocytes and macrophages.

### 4.7 Dynamic Light Scattering

The hydrodynamic diameters and polydispersity indexes (PDIs) of the LNPs were measured using a DynaPro Plate Reader III (Wyatt Technology, Santa Barbara, CA). The LNPs were diluted 10‐fold in 1X PBS‐supplemented DMEM, and 30 µL were loaded onto a 384‐well Aurora plate (Wyatt). The plate was centrifuged for 5 min at 300 g before loading onto the plate reader. Hydrodynamic diameters were measured immediately and after every hour, for 24 h, and are reported as intensity‐weighted averages with *n* = 3 measurements. Data are expressed as mean ± standard deviation, where the standard deviation was calculated by, $\mathrm{STD}=\sqrt{\mathrm{PDI}}\;\times \mathrm{diameter}$. PDI is reported as mean ± standard deviation of the measurements. Data were analyzed using Dynamics 8.1.2.144 (Wyatt Technology).

### 4.8 Cryo‐TEM

Morphology and size were analyzed by cryogenic transmission electron microscopy (cryo‐TEM) by adding 3 µL of the LNPs at an mRNA concentration of 50 ng/µL to a Quantifoil (Jena) holey carbon grid that was glow‐discharged. Grids were blotted and frozen in liquid ethane using a Vitrobot Mark IV (Thermo Fisher Scientific). Imaging was performed at the Beckman Center for Cryo‐EM on a Titan Krios equipped with a K3 Bioquantum (Thermo Fisher Scientific).

### 4.9 ζ‐potential

The ζ‐potential was measured by electrophoretic light scattering using a Zetasizer Ultra (Malvern Instruments, Malvern, UK) by diluting the LNPs 5‐fold in 20 mm Tris pH 7.4 and transferring 1 mL into a DTS1070 capillary sample cell (Malvern).

### 4.10 UV/vis Spectra

The UV/vis spectra of E10i‐494 LNPs containing p53 mRNA or p53 mRNA and 50 mol% CPX were obtained using a NanoDrop ND‐1000 Spectrophotometer (Thermo Fisher Scientific) by first blanking with 1X PBS. Absorbances were obtained every 3 nm from 220 to 748 nm.

### 4.11 Analytical Ultracentrifugation

Sedimentation velocity analytical ultracentrifugation (SV‐AUC) experiments were performed at 20°C using an Optima analytical ultracentrifuge (Beckman Coulter, Brea, CA) equipped with a TiAn50 rotor and two‐channel charcoal‐filled Epon centerpieces with sapphire windows. Data were acquired using both absorbance and interference optics. Samples were prepared in 1X PBS with or without the drug and loaded into leak‐tested SV‐AUC cells. Cells were equilibrated in the instrument at 20°C under full vacuum for 1 h prior to data acquisition. Sedimentation velocity experiments were conducted at 20 000 rpm and 20°C, with radial concentration profiles collected every 30 s. Data were analyzed in SEDFIT (Version 17) using a least‐squares g*(s) distribution model based on direct boundary modeling of the Lamm equation. The sedimentation coefficient distribution was calculated over a range of s = −1000–1000 S with a resolution of 500 grid points. A confidence interval of 0.68 (one standard deviation) was applied to the fit. Tikhonov regularization was employed to stabilize the inversion and suppress amplification of noise while preserving physically meaningful features in the distribution. This approach is particularly important for LNP systems, which can contain both positively sedimenting (dense, RNA‐loaded particles) and negatively buoyant or slowly sedimenting species (e.g., empty or lipid‐rich particles), leading to broad, overlapping, and bidirectional distributions that benefit from controlled smoothing. Time‐invariant and radially invariant noise, as well as time‐dependent noise, were included in the fitting procedure as implemented in SEDFIT. The meniscus position was refined during fitting to determine its optimal value.

### 4.12 In Vitro Assays

To evaluate luciferase expression, cells were plated at 20 000 cells per well in 100 µL of media in tissue culture‐treated white 96‐well plates (Greiner) and were left to adhere for 16 h. Afterward, the media was removed and replaced with the corresponding LNPs encapsulating FLuc mRNA at a concentration of 20 ng of mRNA per well in 100 µL of media. The cells were incubated with the LNPs for 24 h. Luciferase expression was measured by removing the media, adding 50 µL of 1X reporter lysis buffer (Promega, Madison, WI) followed by 100 µL of luciferase assay substrate (Promega). The plate was then wrapped in aluminum foil and then shaken on a plate shaker at 200 rpm for 10 min. Luminescence intensity was quantified using an Infinite 200 Pro plate reader (Tecan) and normalized based on the average intensity of the C12‐200 LNP. Normalized luminescence is reported as mean ± SD of *n* = 3 biological replicates, each averaged from *n* = 4 technical replicates. Cellular toxicity of the LNPs was measured using the same cell setup as described above, where LNP dose depended on the specific experiment. After incubating the cells with LNPs for 24 or 48 h, 100 µL of CellTiter‐Glo (Promega) was added to each well, and then the plate was wrapped in aluminum foil and shaken on a plate shaker at 200 rpm for 10 min. The luminescence corresponding to ATP concentration was quantified using an Infinite 200 Pro plate reader and normalized by dividing the luminescence from each group by the average luminescence signal of an untreated group. Cell viability percentage was reported as mean ± SD of *n* = 3 biological replicates, each averaged from *n* = 3 technical replicates.

Caspase‐3/7 assays were performed using the AnaSpec (Fremont, CA, USA) SensoLyte Homogenous AMC Caspase‐3/7 Fluorimetric Assay Kit. Tissue‐treated black 96‐well plates (Greiner) containing 20 000 cells per well were prepared as described above. After incubating LNPs at 5, 20, and 50 ng of mRNA per well for 24 h in a total volume of 150 µL, 50 µL of the Caspase‐3/7 substrate solution was added to each well, and the plate was wrapped in aluminum foil and shaken on a plate shaker at 200 rpm for 1 min. Fluorescence corresponding to caspase‐3/7 activity was quantified using an Infinite 200 Pro plate reader using an excitation wavelength of 354 nm and emission wavelength of 442 nm. Fluorescence was normalized by dividing the fluorescence signal from each group by the average fluorescence signal of an untreated group. RFU was reported as mean ± SD of *n* = 4 technical replicates.

Caspase‐9 assays were performed using the Promega Caspase‐Glo 9 Assay System. Tissue‐treated white 96‐well plates containing 20 000 cells per well were prepared as described above. After incubating LNPs at 50 ng of mRNA per well for 24 h, 100 µL of Caspase‐Glo 9 Reagent was added to each well and the plate was wrapped in aluminum foil and shaken on a plate shaker at 300 rpm for 2 min. The plate was then removed from the shaker and incubated at room temperature for 30 min. Luminescence corresponding to caspase‐9 activity was quantified using an Infinite 200 Pro plate reader and normalized by dividing the luminescence from each group by the average luminescence signal of an untreated group. Relative luminescence was reported as mean ± SD of *n* = 3 technical replicates.

Mitochondrial permeability assays were performed using the APExBio Mitochondrial Permeability Transition Pore Assay Kit. Tissue‐treated black 96‐well plates containing 20 000 CAL‐27 cells per well were prepared as described above. LNPs were added to the cells at 5, 20, and 50 ng of mRNA per well for 24 h in a total volume of 100 µL. After 24 h, the Calcein AM working solution and fluorescence quenching working solution were added, and the cells were incubated at 37°C for 30 min before being washed with dilution buffer. Fluorescence corresponding to mitochondrial permeability was quantified using an Infinite 200 Pro plate reader using an excitation wavelength of 485 nm and an emission wavelength of 525 nm. Fluorescence was normalized by dividing the fluorescence signal from each group by the average fluorescence signal of an untreated group. RFU was reported as mean ± SD of *n* = 4 technical replicates.

Reactive oxygen species (ROS) assays were performed using the Thermo Fisher Scientific Total Reactive Oxygen Species Assay Kit (520 nm). Tissue‐treated black 96‐well plates containing 20 000 CAL‐27 cells per well were prepared as described above. After adhering, the ROS Assay Stain was added to the cells, and the cells were incubated at 37°C for 60 min. LNPs were added to the cells at 5, 20, and 50 ng of mRNA per well for 24 h in a total volume of 100 µL. After 24 h, fluorescence corresponding to ROS activity was quantified using an Infinite 200 Pro plate reader using an excitation wavelength of 480 nm and emission wavelength of 520 nm. Fluorescence was normalized by dividing the fluorescence signal from each group by the average fluorescence signal of an untreated group. RFU was reported as mean ± SD of *n* = 4 technical replicates.

Griess assays were performed using the Promega Griess Reagent System. TAMs were plated on non‐tissue‐treated black 96‐well plates at a density of 100 000 cells per well. Upon adhering for 16 h, LNPs were added at a dose of 100 ng of mRNA per well. After an additional 24 h, 50 µL of media from each well was transferred to a clear 96‐well plate (Thermo Fisher Scientific). A nitrite standard curve was prepared according to the manufacturer's instructions using concentrations ranging from 100 to 1.56 µm and included a blank well. Then, 50 µL of the sulfanilamide solution was added to each well. The plate was incubated for 5 min at room temperature away from light, before the addition of 50 µL of the NED solution to all wells. The plate was incubated for another 5 min at room temperature, protected from light. Absorbance corresponding to nitrite concentration was measured at 535 nm using an Infinite 200 Pro plate reader. Nitrite concentration was determined via a standard curve estimated from an LSLR. Nitrite concentration was reported as mean ± SD of *n* = 4 technical replicates.

### 4.13 In Vitro Flow Cytometry

For dose response curves, E10i‐494 LNP was formulated with mCherry mRNA. Cancer cells were plated in tissue‐treated 24‐well plates (Greiner) while M0 macrophages and TAMs were plated in non‐tissue‐treated 24‐well plates (Thermo Fisher Scientific) at cell densities of 250 000 cells per well. The cells adhered for 16 h, and then mCherry‐containing E10i‐494 was added to each well at mRNA doses ranging from 0.05 to 250 ng for cancer cells and 5 ng to 1,000 ng for M0 macrophages and TAMs. After 24 h of LNP incubation, the media was removed, and the cells were washed with 1X PBS. The cancer cells were removed with 0.25% Trypsin‐EDTA (Thermo Fisher Scientific) while the M0 macrophages and TAMs were removed with TrypLE Express (Gibco) followed by scraping with 40 cm cell scrapers (Greiner). After detachment, the oral cancer cells were diluted 2‐fold in supplemented DMEM, while the M0 macrophages and TAMs were diluted 2‐fold in their corresponding media. The cells were centrifuged at 400 g for 5 min, where the supernatant was aspirated, and the cells were resuspended in 1X PBS before being analyzed on a FACSymphony A5 SE cell analyzer (Becton Dickinson, Franklin Lakes, NJ). mCherry+ cells were reported as mean ± SD of *n* = 3 technical replicates.

For endosomal escape analysis, E10i‐494 LNPs with no CPX, 25 mol% CPX, or 50 mol% CPX were reformulated with 1,1'‐dioctadecyl‐3,3,3',3'‐tetramethylindotricarbocyanine iodide (DiR) and Cy5‐tagged GFP mRNA. DiR was incorporated into the ethanol phase during formulation at 0.5 mol%, subtracting from the ionizable lipid contribution. LNPs were dosed at 10 ng (low dose) or 100 ng (high dose) per 200 000 cells. Flow cytometry was performed as described above.

For TAM phenotype experiments, TAMs were plated as described above and treated with LNPs at a dose of 250 ng of mRNA per well. After detaching and resuspending in 1X PBS, TAMs were extracellularly stained for 30 min with BV421‐CD163 (BioLegend, San Diego, CA, USA), APC‐CD38 (BioLegend), BV510‐CD274 (BioLegend), and PE/Cy5‐CD86 (BioLegend). The cells were then fixed, permeabilized, and stained intracellularly with AF488‐Arg‐1 (Thermo Fisher Scientific), PE‐CD206 (Becton Dickson), APC/Cy7‐CD68 (BioLegend), and AF594‐p53 (Santa Cruz Biotechnology, Dallas, TX, USA) using the Cyto‐Fast Fix/Perm Buffer Set (BioLegend) according to the manufacturer's instructions. TAMs were resuspended in 1X PBS before being analyzed on a FACSymphony A5 SE cell analyzer. Median fluorescent intensity (MFI) was reported as mean ± SD of *n* = 5 technical replicates.

### 4.14 Murine Tumor Burden Studies With CAL‐27 Cells

Nu/J male mice of 6–8 weeks old with an average weight of 25 g were purchased from Jackson Laboratory (Bar Harbor, ME). Animals were housed in a barrier facility with air humidity 40%–70%, ambient temperature of 22 ± 2°C, and 12 h dark/light cycle. The mice were inoculated on the right flank with two million CAL‐27 cells suspended in 1X cold PBS and 25% Matrigel (Corning, Corning, NY, USA). After 2 weeks, for biodistribution studies LNPs encapsulating FLuc mRNA were intratumorally injected into the tumors at a dose of 0.1 mg of encapsulated mRNA per kg of body weight (mpk) using a total volume of 20 µL. After 6 h, the mice were intraperitoneally injected with D‐luciferin (0.2 mL, 15 mg/mL; Biotium, Fremont, CA, USA). After 5 min, the mice were euthanized by CO_2_ asphyxiation followed by cervical dislocation. Then, the heart, lungs, liver, kidneys, spleen, and tumor were dissected and imaged using an In Vivo Imaging System (IVIS; Revvity, Waltham, MA, USA). Total flux was quantified by the Living Image Software 4.7.3 (PerkinElmer) by placing rectangular regions of interest (ROI) around the organ and tumor images, keeping the same ROI sizes for each organ and tumor. Total flux was reported as mean ± SD of *n* = 3 biological replicates.

For tumor burden, mice were inoculated as described above, where each mouse received subcutaneous injections of CAL‐27 cells on the left and right flanks. Two weeks after inoculation, LNPs were injected intratumorally four times over the course of 10 days at 0.1 mpk using a total volume of 20 µL in each tumor. Tumor volume was monitored using calipers and calculated as 0.5 × width^2^ × height. After 18 days from the first dose, the mice were sacrificed, and tumors were dissected for further immunohistochemistry (IHC) analysis for the protein expression of human p53 and human PUMA genes. Slides were prepared by the Histotechnology Facility at the Wistar Institute. Images were acquired using an EVOS M7000 Imaging System (Thermo Fisher Scientific). Fiji/ImageJ v1.54j was utilized to process IHC slides through the plug‐in Colour Deconvolution2, selecting “H DAB” to separate out the stained protein of interest. Percent area is measured from *n* = 10 images, with a solid representing the median and dashed lines representing quartiles.

### 4.15 Syngeneic Murine Tumor Burden Studies With MOC‐2 OSCC Cells

C57BL/6J female mice of 6–8 weeks old with an average weight of 20 g were purchased from Jackson Laboratory. The fur on the right flank was shaved, and then the flank was inoculated with 200 000 MOC‐2 cells suspended in 0.1 mL of 1X cold PBS containing 25% Matrigel. After 7 days, LNPs were injected intratumorally four times over the course of 10 days at 1.0 mpk using a total volume of 20 µL. Tumor volume was measured for up to 40 days after the initial dose. For toxicity evaluation and immune cell infiltration analysis in the TME, C57BL/6J mice were inoculated as described above. After 14 days, LNPs were injected intratumorally three times over the course of 4 days at 1.0 mpk using a total volume of 20 µL. After a total of 6 days from the first LNP injection, blood was collected, and then the mice were sacrificed, and the tumors were dissected for IHC and flow cytometry.

For IHC, tumor tissues were fixed with 4% PFA (MilliporeSigma), and paraffin‐embedded 5 µm sections were deparaffinized with xylene (MilliporeSigma), rehydrated with 200 proof ethanol (Thermo Fisher Scientific), and heated in 10 mmol/L sodium citrate buffer (MilliporeSigma) at pH 6.0 for antigen retrieval. After blocking with 2.5% goat serum (Thermo Fisher Scientific) in 1X PBS, the sections were incubated for 16 h at 4 °C with primary antibodies, then detected using a universal immunoperoxidase ABC kit. All the sections were counterstained with hematoxylin. Images were acquired and quantified as described above. Percent area is measured from *n* = 8 images, with a solid line representing the median and dashed lines representing quartiles.

To assess toxicity, blood was collected by retro‐orbital bleeding in Microtainer blood collection tubes containing serum separator gel (BD Biosciences). The tubes were centrifuged for 10 min at 2000 g , and serum was isolated. Serum levels of AST, ALT, and creatinine were determined using colorimetric activity assays, while the serum urea level was determined using a fluorometric assay (Cayman Chemical, Ann Arbor, MI, USA).

For flow cytometry, the tumors were diced and incubated in digestion medium containing DNase I (New England Biolabs; Ipswich, MA), collagenase IV (Thermo Fisher Scientific), and dispase II (Fisher Scientific) for 1 h at 37°C. Afterward, the solution was passed through a 70 µm filter (Corning) into 15 mL centrifugal tubes (Corning). The tubes were centrifuged at 500 g for 5 min. The supernatant was removed, and the cells were resuspended in ACK Lysis Buffer (Thermo Fisher Scientific) on ice for 5 min. Afterward, the solution was diluted 2.5‐fold in FACS buffer containing 0.6% BSA, 25 mm 4‐(2‐hydroxyethyl)‐1‐piperazineethanesulfonic acid (HEPES; Thermo Fisher Scientific), and 1 mM ethylenediaminetetraacetic acid (EDTA; Thermo Fisher Scientific) in 1X PBS. The cells were then centrifuged at 500 g for 5 min, and the supernatant was aspirated. Then, the cells were stained with Live/Dead Zombie Aqua (BioLegend) according to the manufacturer's instructions, followed by extracellular staining with BV605‐CD163 (BioLegend), Spark UV 387‐CD4 (BioLegend), FITC‐F4/80 (BioLegend), APC/Fire750‐CD11b (BioLegend), AF700‐CD45 (BioLegend), BV785‐CD3 (BioLegend), BUV805‐CD274 (BioLegend), PE‐Dazzle594‐CD8a (BioLegend), PE‐Cy7‐CD68 (BioLegend), and APC‐CD38 (BioLegend). The cells were then fixed, permeabilized, and stained intracellularly with PE‐CD206 (BioLegend) and AF488‐Arg‐1 (Thermo Fisher Scientific) using the Cyto‐Fast Fix/Perm Buffer Set (BioLegend) according to the manufacturer's instructions. The cells were resuspended in FACS buffer before being analyzed on a FACSymphony A5 SE cell analyzer. Median fluorescent intensity (MFI) was reported as mean ± SD of *n* = 14 for PBS, *n* = 10 for p53‐LNP, *n* = 9 for FLuc‐LNP and CPX/p53‐LNP, and *n* = 6 for CPX‐LNP.

### 4.16 Statistical & Reproducibility

All statistical analyses were performed in GraphPad Prism Version 10.5.0 (GraphPad Software, Inc, La Jolla, USA). All tests of significance were performed at a significance level of α = 0.05. For experiments with one variable where multiple technical or biological replicates were performed, one‐way analyses of variance (ANOVAs) with post hoc Holm–Šídák correction for multiple comparisons were used to compare responses across treatment groups. For experiments that measured two variables with more than two treatment groups, a two‐way ANOVA with post hoc Holm–Šídák correction for multiple comparisons was used to compare responses across treatment groups. For experiments that measured two variables with two treatment groups in each variable, two‐tailed multiple unpaired *t* tests with Holm–Šídák correction for multiple comparisons were used to compare responses across treatment groups. Survival curves were compared by log‐rank (Mantel–Cox) tests with Holm–Šídák correction for multiple comparisons. All data are presented as mean ± standard deviation unless otherwise reported. No statistical method was used to predetermine sample size. No data were excluded from the analyses. The experiments were not randomized. The investigators were not blinded to allocation during experiments and outcome assessment.

## Author Contributions

M.S.P., Q.Z., A.D.L., and M.J.M. designed the experiments. M.S.P., J.J.L., Q.Z., K.P., S.S., H.M.Y., R.A.J., B.N.H., O.Z.C., E.F., K.G. and S.V.T. performed the experiments. M.S.P., J.J.L., Q.Z., K.P., and S.V.T. analyzed the data. M.S.P. wrote the manuscript. Q.Z., A.D.L., M‐G.A., and D.W. edited the manuscript. All authors discussed the results and commented on the manuscript.

## Conflicts of Interest

M.S.P. and M.J.M. have a patent related to the structure of the BEND lipids and their biological applications (U.S. Provisional Patent Appl. No. 63/373,793, filed August 29, 2022).

## Supporting information




**Supporting File**: adma73721‐sup‐0001‐SuppMat.pdf.

## Data Availability

All relevant data supporting the findings of this study are available within the paper and Supplementary Information.
