# Supplementary material for: Lipid Nanoparticle Co‐Delivery of mRNA and a Small Molecule Drug for Oral Cancer Chemoimmunotherapy
Source: Adv Mater. 2026 Jul 14;38(47):e73721. doi: 10.1002/adma.73721 (PMC13496076; doi:10.1002/adma.73721)
Supplement: Supplementary file 1 [file ADMA-38-e73721-s001.pdf]

Supplementary Information

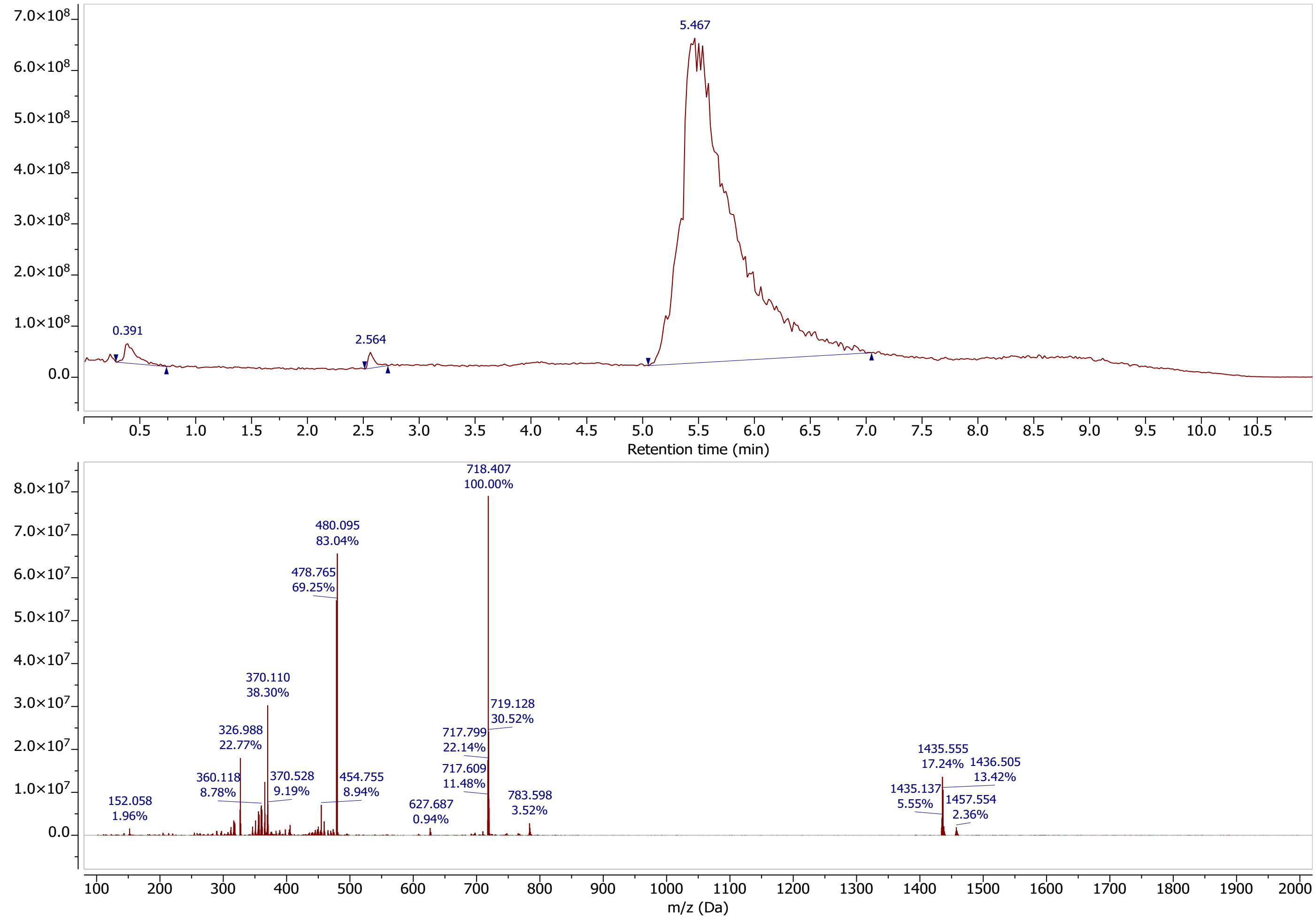

Supplementary Figure 1. LC-MS trace of E10i-494. Electrospray ionization mass spectrometry calculated for E10i-494, 1435.47; found 1435.56. Purity: 97.94%

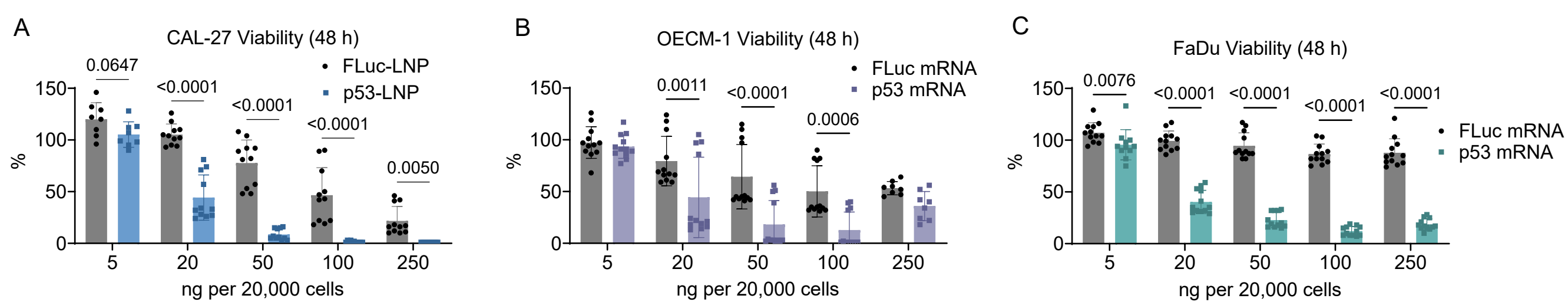

Supplementary Figure 2. In vitro toxicity of p53-mRNA-loaded LNPs after 48 h. A–C, E10i-494 was formulated with FLuc mRNA (FLuc-LNP) and human p53 mRNA (p53-LNP). The two LNPs were incubated in **(A)** CAL-27, **(B)** OECM-1, and **(C)** FaDu cells at increasing dosages per 20,000 cells, and after 48 h, viability was measured. Viability is reported as mean ± SD of n = 9–12. Multiple unpaired t test with *post hoc* Holm–Šídák correction for multiple comparisons was used to compare FLuc-LNP to p53-LNP.

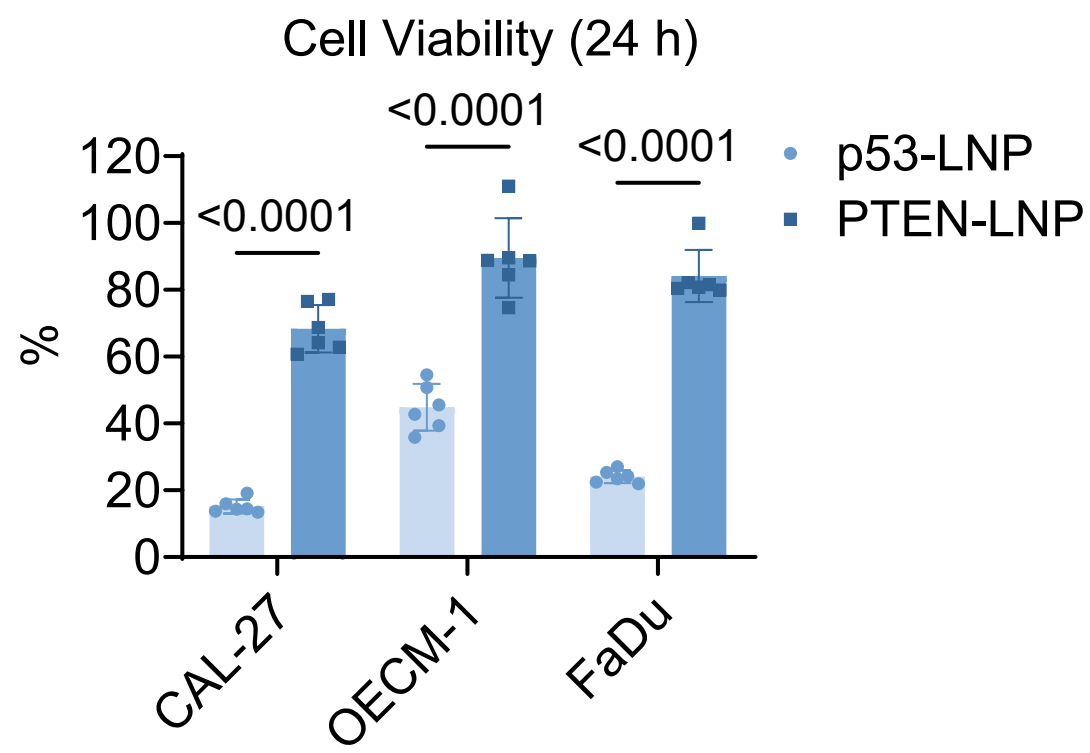

Supplementary Figure 3. Comparison between LNPs encapsulating PTEN mRNA and p53 mRNA. E10i-494 encapsulating human p53 mRNA (p53-LNP) and PTEN mRNA (PTEN-LNP) were dosed in CAL-27, OECM-1, and FaDu at 20 ng per 20,000 cells, and after 24 h, cellular viability was measured. Viability is reported as mean ± SD of *n* = 6. Multiple unpaired t test with *post hoc* Holm–Šídák correction for multiple comparisons was used to compare p53 mRNA to PTEN mRNA.

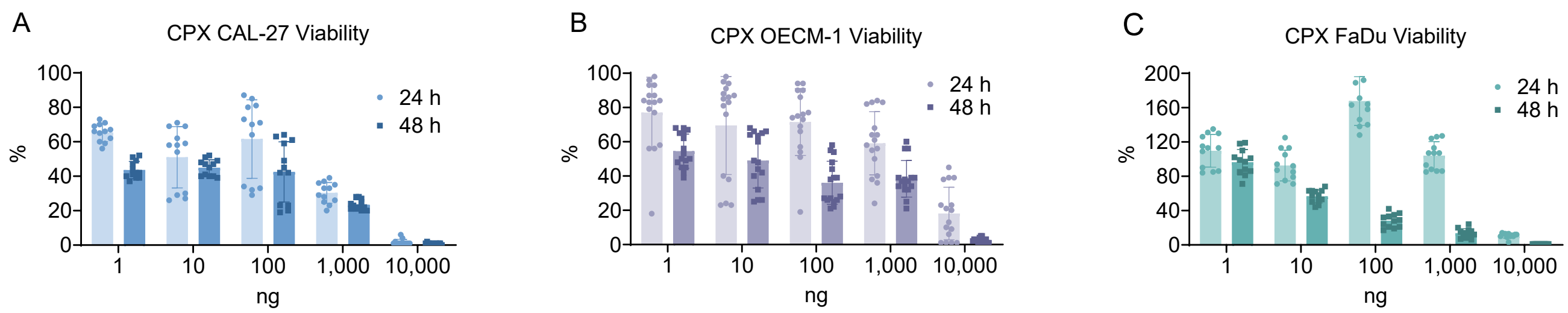

Supplementary Figure 4. CPX chemotherapeutic efficacy in OSCC lines. A–C, Free CPX was incubated with **(A)** CAL-27, **(B)** OECM-1, and **(C)** FaDu cells at increasing doses per 20,000 cells. After 24 and 48 h, cellular viability was measured. Viability is reported as mean ± SD of *n* = 12.

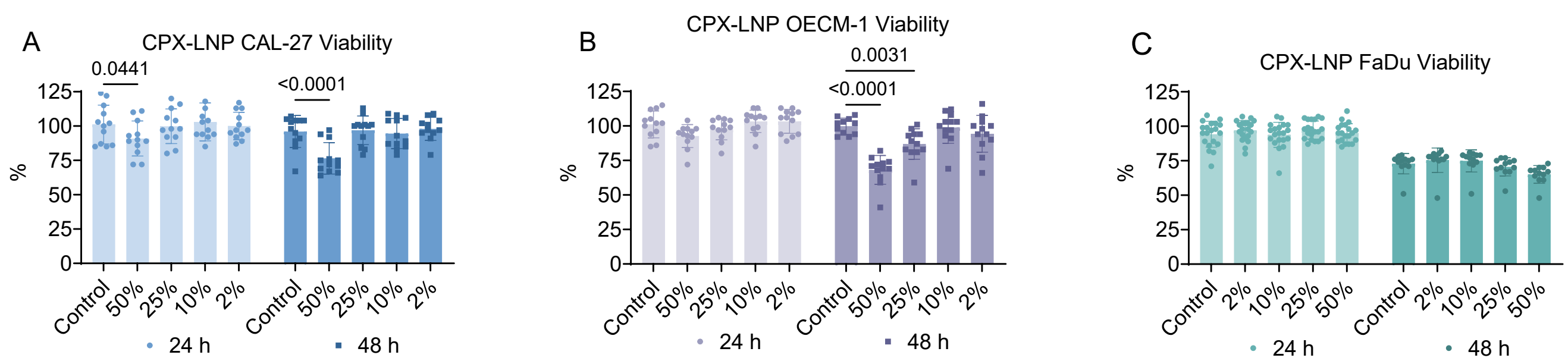

Supplementary Figure 5. CPX-LNP toxicity in OSCC lines. A–C, E10i-494 encapsulating increasing mol% of CPX were dosed in **(A)** CAL-27, **(B)** OECM-1, and **(C)** FaDu at 20 ng per 20,000 cells, and after 24 and 48 h, cellular viability was measured. Control formulation has no CPX. Viability is reported as mean ± SD of *n* = 12. Two-way ANOVA with *post hoc* Holm–Šídák correction for multiple comparisons was used to compare the control LNP against all other formulations for each time point. No comparisons reached significance for FaDu cells.

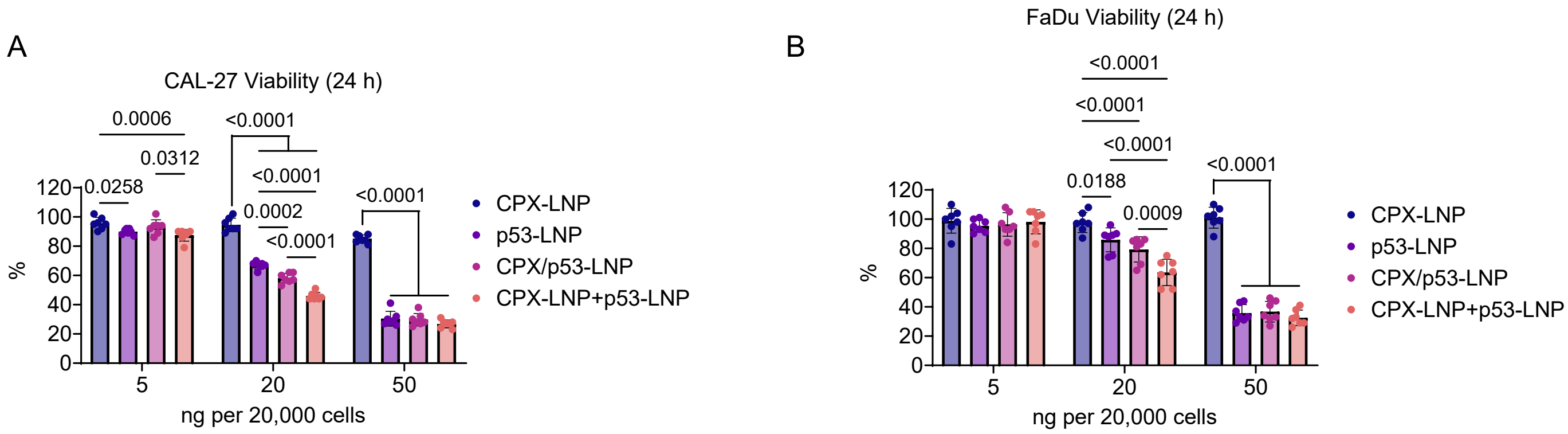

Supplementary Figure 6. Comparison of co-formulated CPX and p53 mRNA with separate LNPs. A–B, E10i-494 encapsulating CPX at 50 mol% (CPX-LNP), p53 mRNA (p53-LNP), or both compounds (CPX/p53-LNP) were formulated. The separate LNPs as well as a 1:1 (mass of mRNA) combination of p53-LNP and CPX-LNP (CPX-LNP+p53-LNP) were dosed in **(A)** CAL-27 and **(B)** FaDu at 5, 20, and 50 ng per 20,000 cells, and after 24 h, cellular viability was measured. For CPX-LNP+p53-LNP, dose is based on encapsulated p53 mRNA only. Viability is reported as mean ± SD of *n* = 7. Two-way ANOVA with *post hoc* Holm–Šídák correction for multiple comparisons was used to compare formulations at each dose.

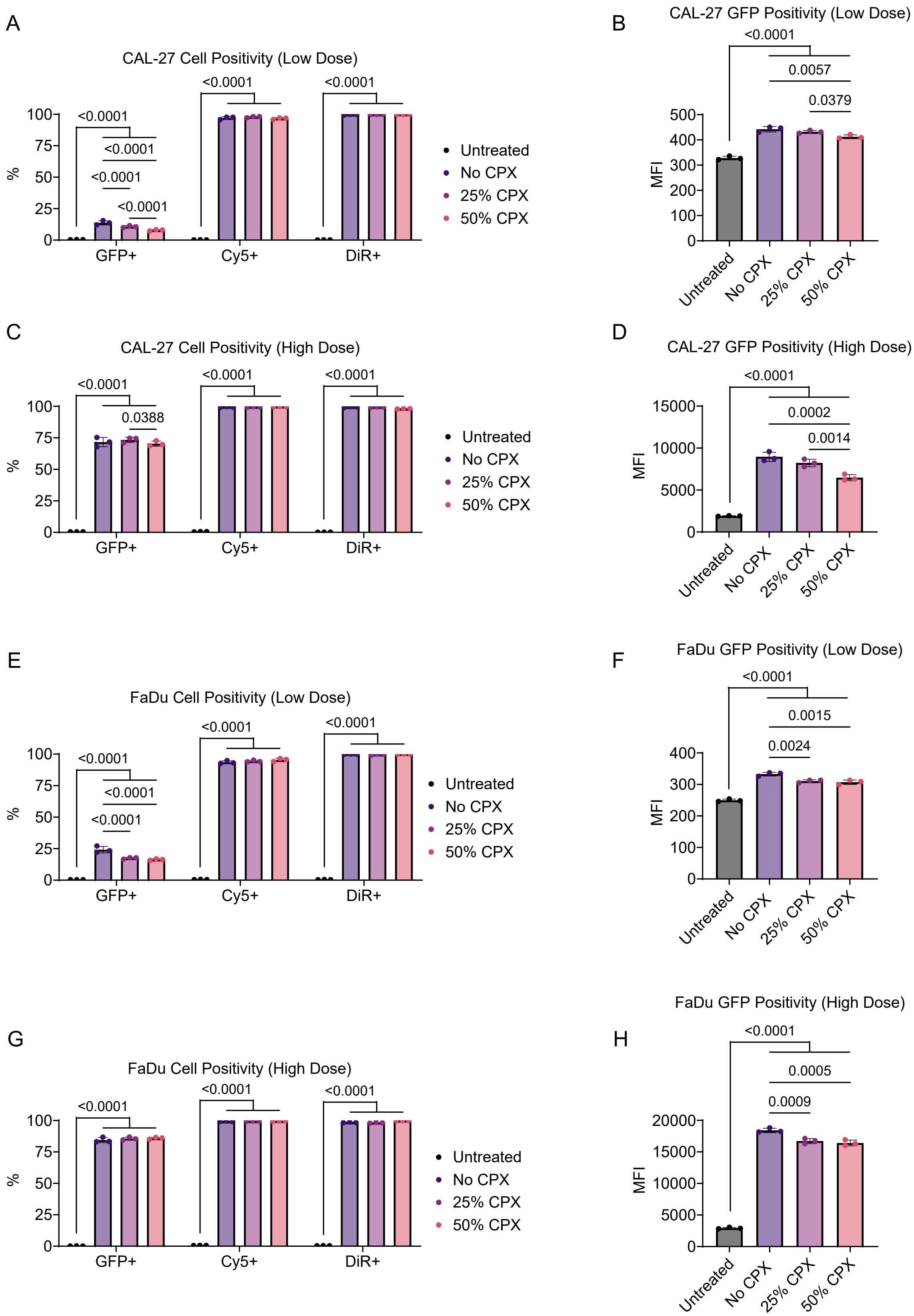

Supplementary Figure 7. Role of CPX in LNP and mRNA cellular accumulation. A–H, E10i-494 encapsulating Cy5-tagged green fluorescent protein (GFP) mRNA and 0.5 mol% DiR were formulated with 0% CPX, 25% CPX, and 50% CPX and dosed in CAL-27 and FaDu cells at 10 ng (low dose) or 100 ng (high dose) per 200,000 cells. After 24 h, **(A)** low dose cell positivity in CAL-27 cells, **(B)** low dose GFP MFI in CAL-27 cells, **(C)** high dose cell positivity in CAL-27 cells, **(D)** high dose GFP MFI in CAL-27 cells, **(E)** low dose cell positivity in FaDu cells, **(F)** low dose GFP MFI in FaDu cells, **(G)** high dose cell positivity in FaDu cells, **(H)** high dose GFP MFI in FaDu cells were analyzed by flow cytometry. Percent transfection and MFI are reported as mean ± SD of n = 3. **A, C, E, G,** Two-way

ANOVA with *post hoc* Holm–Šídák correction for multiple comparisons was used to compare formulations for each fluorophore. **B, D, F, H,** One-way ANOVA with *post hoc* Holm–Šídák correction for multiple comparisons was used to compare GFP MFI across each formulation.

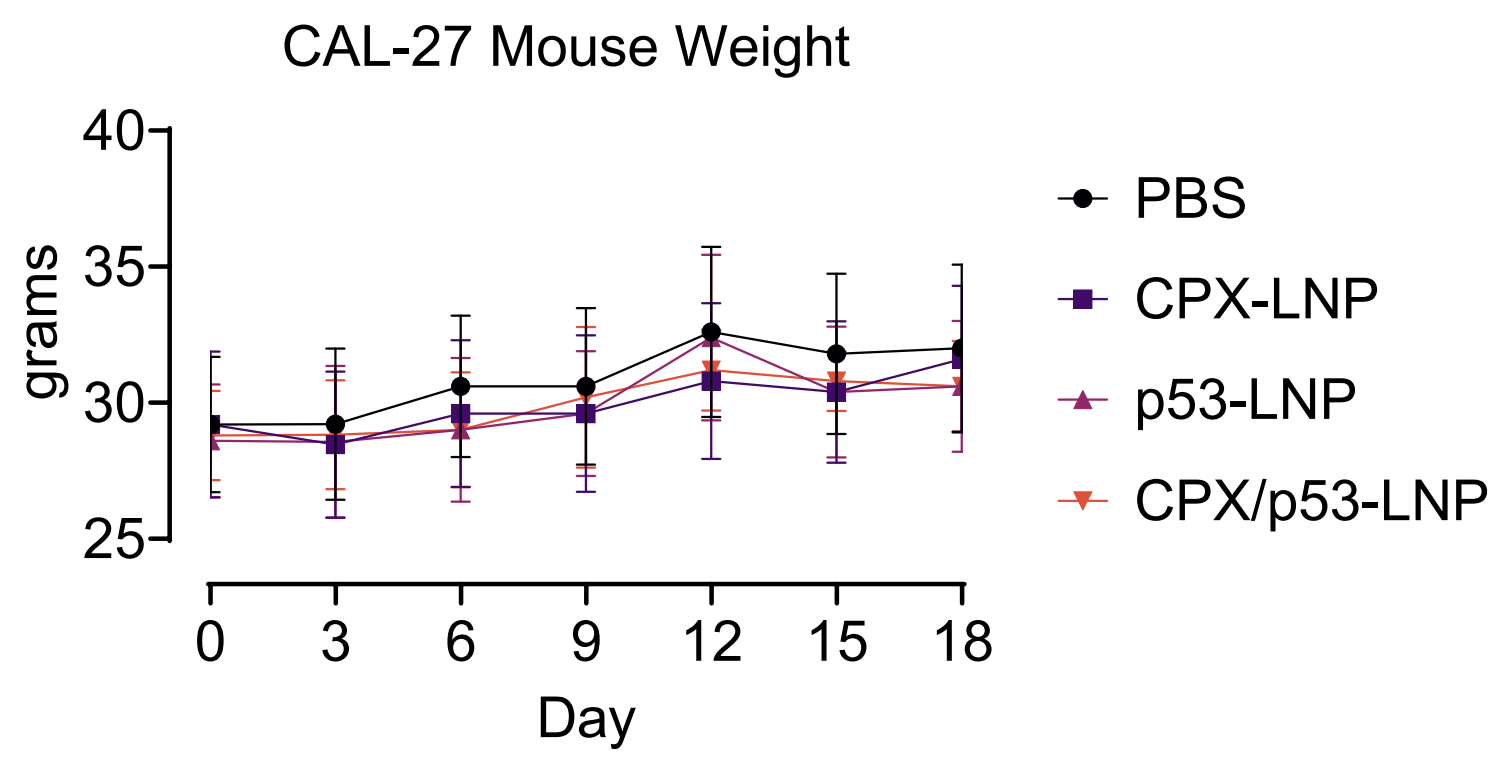

Supplementary Figure 8. Mouse weights for CAL-27 xenograft study. Nu/J mice were inoculated with two million CAL-27 cells on each flank, and after two weeks, LNPs were administered intratumorally at an mRNA dose of 0.1 mg per kg (mpk) with tumors on each flank receiving four doses over ten days. Mouse weight was monitored from the initial dose on day 0 until day 18. Weight is reported as mean ± SD of n = 5.

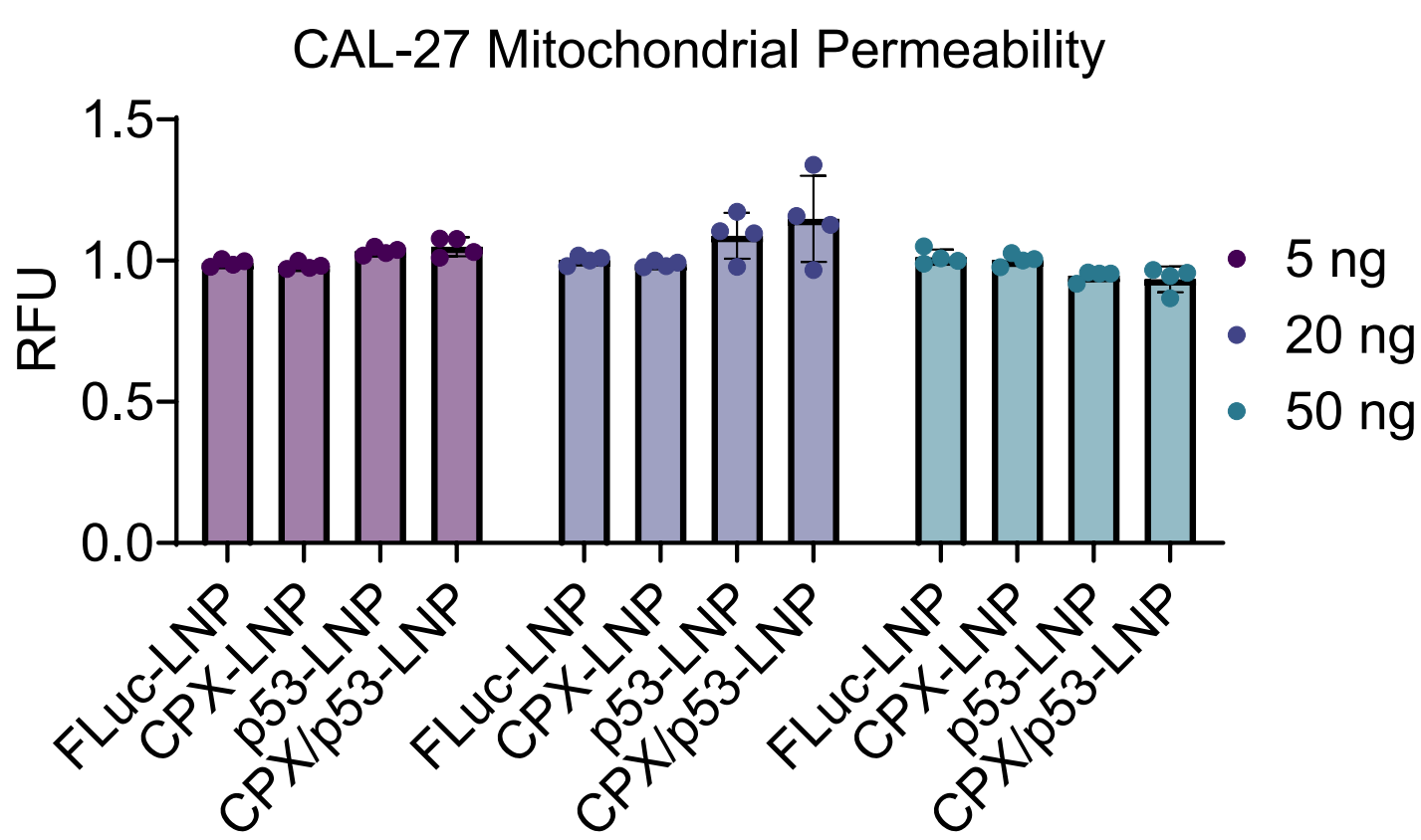

Supplementary Figure 9. Mitochondrial permeability assay. E10i-494 LNPs containing FLuc mRNA (FLuc-LNP), 50 mol% CPX (CPX-LNP), human p53 mRNA (p53-LNP), and 50 mol% CPX and human p53 (CPX/p53-LNP) were dosed in CAL-27 cells at 5, 20, and 50 ng per 20,000 cells. After 24 h, a Calcein AM-based mitochondrial permeability assay was performed, where fluorescence was calculated relative to untreated cells. Relative fluorescent units (RFU) are reported as mean ± SD of n = 4. Two-way ANOVA with *post hoc* Holm–Šídák correction for multiple comparisons was used to compare individual LNPs for each dose. No comparisons reached significance.

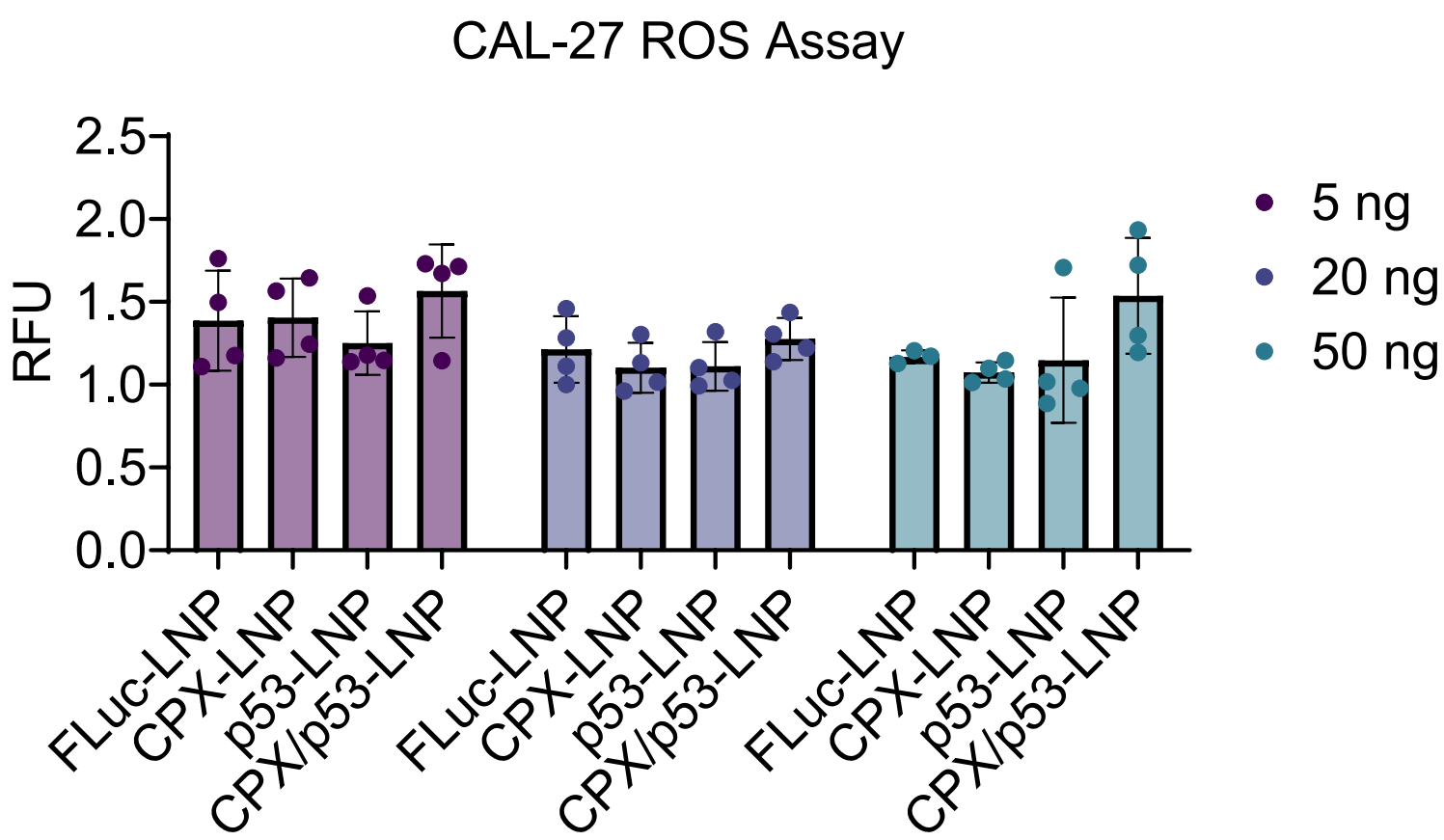

Supplementary Figure 10. ROS assay. E10i-494 LNPs containing FLuc mRNA (FLuc-LNP), 50 mol% CPX (CPX-LNP), human p53 mRNA (p53-LNP), and 50 mol% CPX and human p53 (CPX/p53-LNP) were dosed in CAL-27 cells at 5, 20, and 50 ng per 20,000 cells. After 24 h, a fluorescence-based reactive oxygen species (ROS) assay was performed, where fluorescence was calculated relative to untreated cells. Relative fluorescent units (RFU) are reported as mean ± SD of n = 4. Two-way ANOVA with *post hoc* Holm–Šídák correction for multiple comparisons was used to compare against all other formulations. No comparisons reached significance.

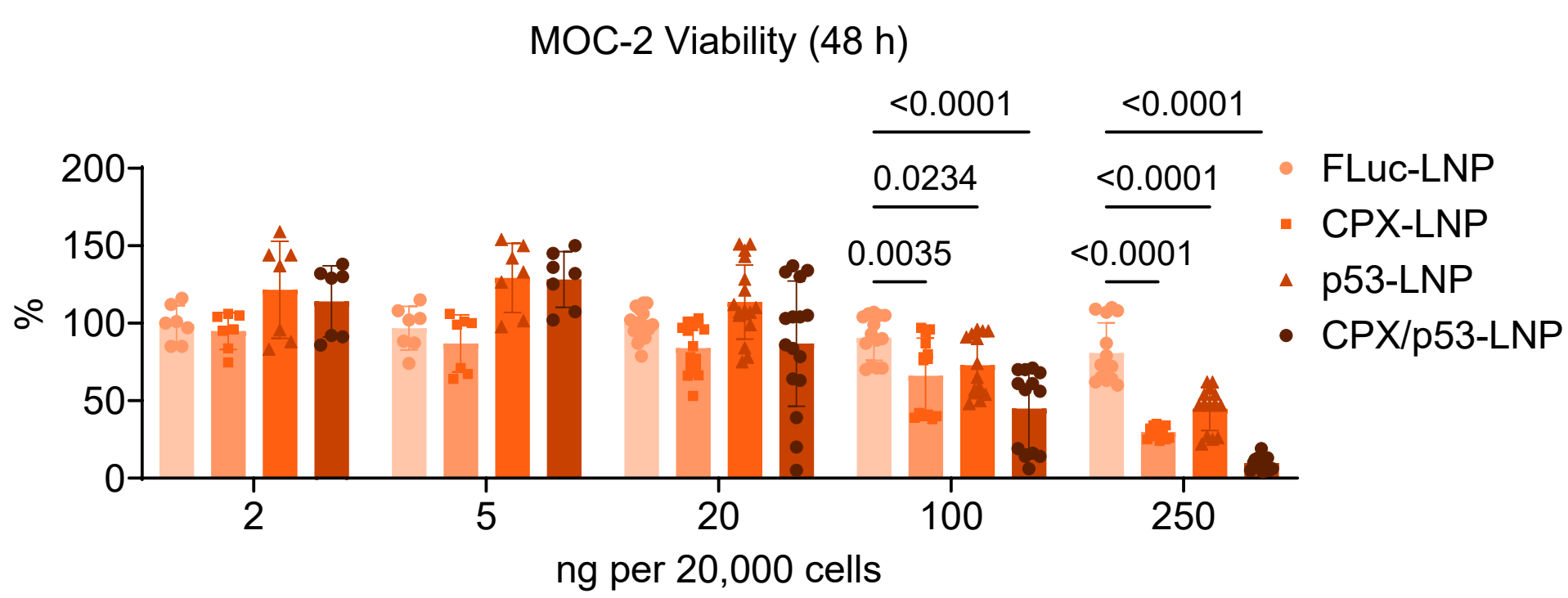

Supplementary Figure 11. MOC-2 viability after 48 h of therapeutic mRNA treatment. E10i-494 LNPs containing FLuc mRNA (FLuc-LNP), 50 mol% CPX (CPX-LNP), mouse p53 mRNA (p53-LNP), and 50 mol% CPX and mouse p53 (CPX/p53-LNP) were incubated in MOC-2 cells at increasing doses per 20,000 cells, and after 48 h, viability was measured. Cellular viability is reported as mean ± SD of n = 8 for 2 ng and 5 ng doses and n = 12 for all other doses. Two-way ANOVA with *post hoc* Holm–Šídák correction for multiple comparisons was used to compare the FLuc-LNP against all other formulations for each dose.

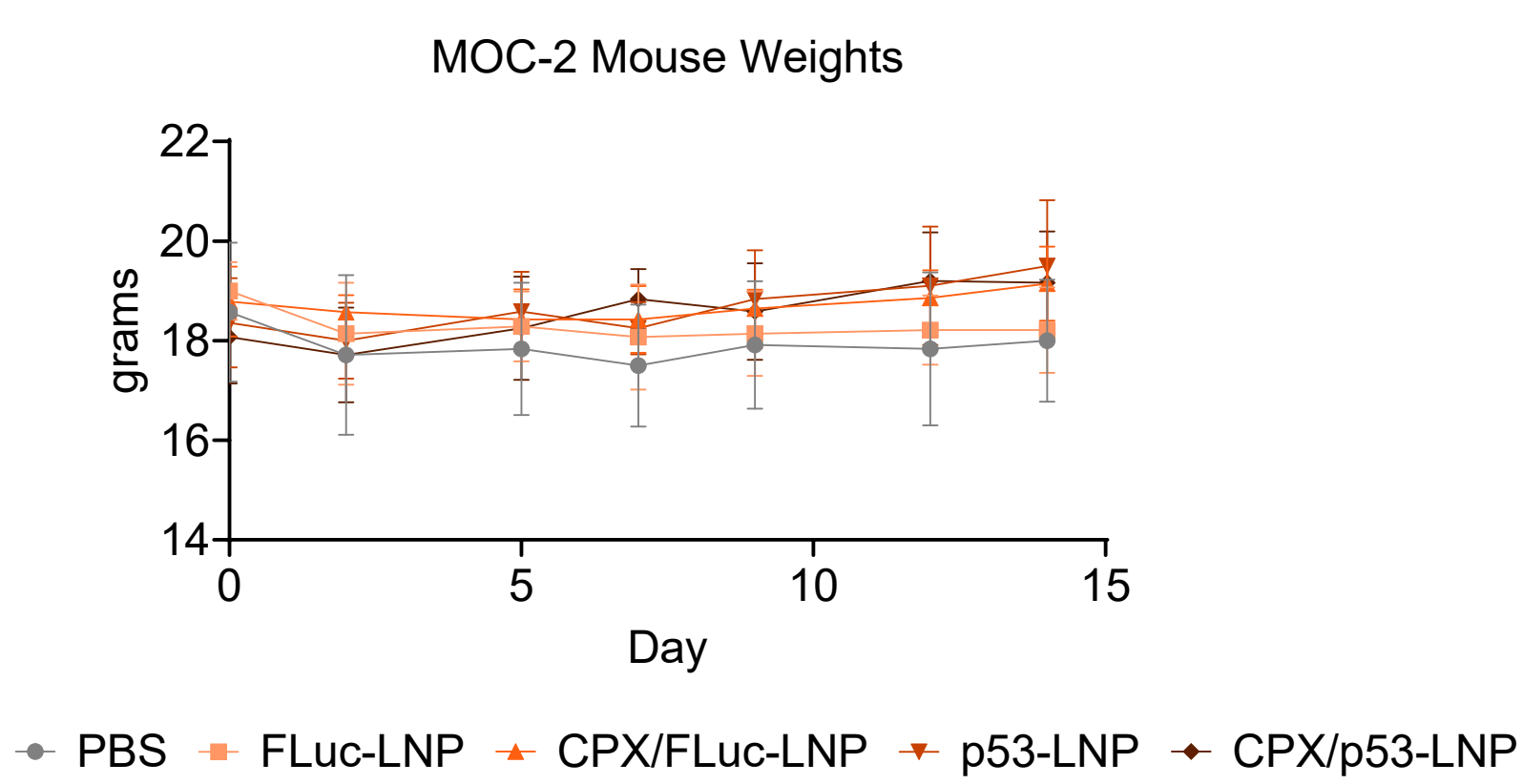

Supplementary Figure 12. Mouse weights for MOC-2 survival study. C57BL/6J mice were inoculated with 200,000 MOC-2 cells on the right flank, and after seven days, LNPs were administered intratumorally at an mRNA

dose of 1.0 mpk four times over ten days. Mouse weight was monitored from the initial dose on day 0 until day 14. Weight is reported as mean ± SD of n = 6 for PBS and n = 5 for all other groups.

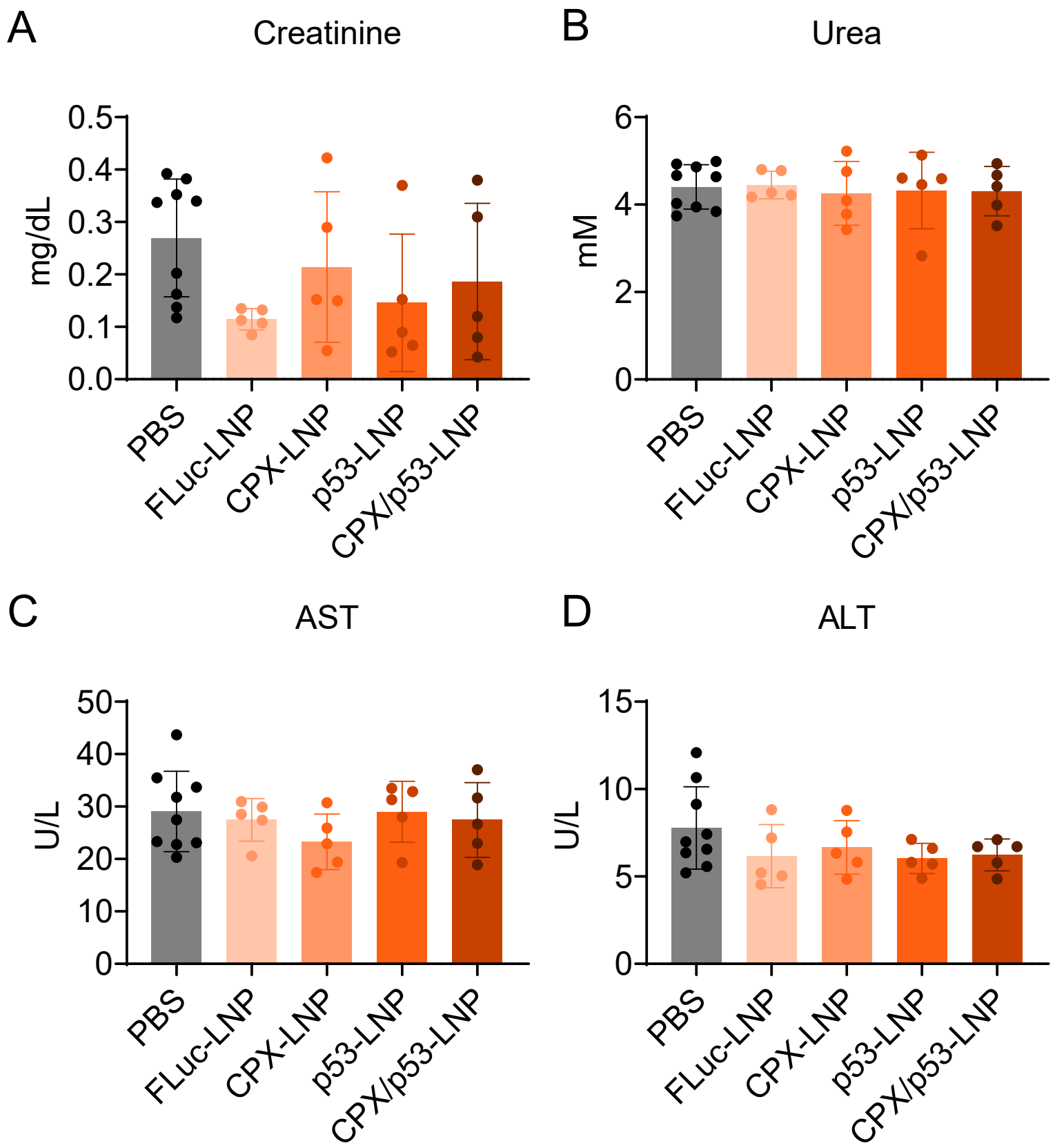

Supplementary Figure 13. Analysis of systemic toxicity markers in a MOC-2 syngeneic model. A–D, E10i-494 LNPs encapsulating firefly luciferase (FLuc-LNP), 50 mol% CPX (CPX-LNP), murine p53 mRNA (p53-LNP), 50 mol% CPX and murine p53 mRNA (CPX/p53-LNP), and PBS were injected three times over four days intratumorally into C57BL/6J mice inoculated with MOC-2 cells. After 48 h from the third dose, blood was taken retro-orbitally and serum was isolated. From the serum, **(A)** creatinine, **(B)** urea, **(C)** aspartate aminotransferase (AST), and **(D)** alanine aminotransferase (ALT) levels were quantified using colorimetric or fluorometric kits. Values are reported as mean ± SD of n = 9 for PBS and n = 5 for all other groups. One-way ANOVA with *post hoc* Holm–Šídák correction for multiple comparisons was used to compare formulations. No comparisons reached significance.

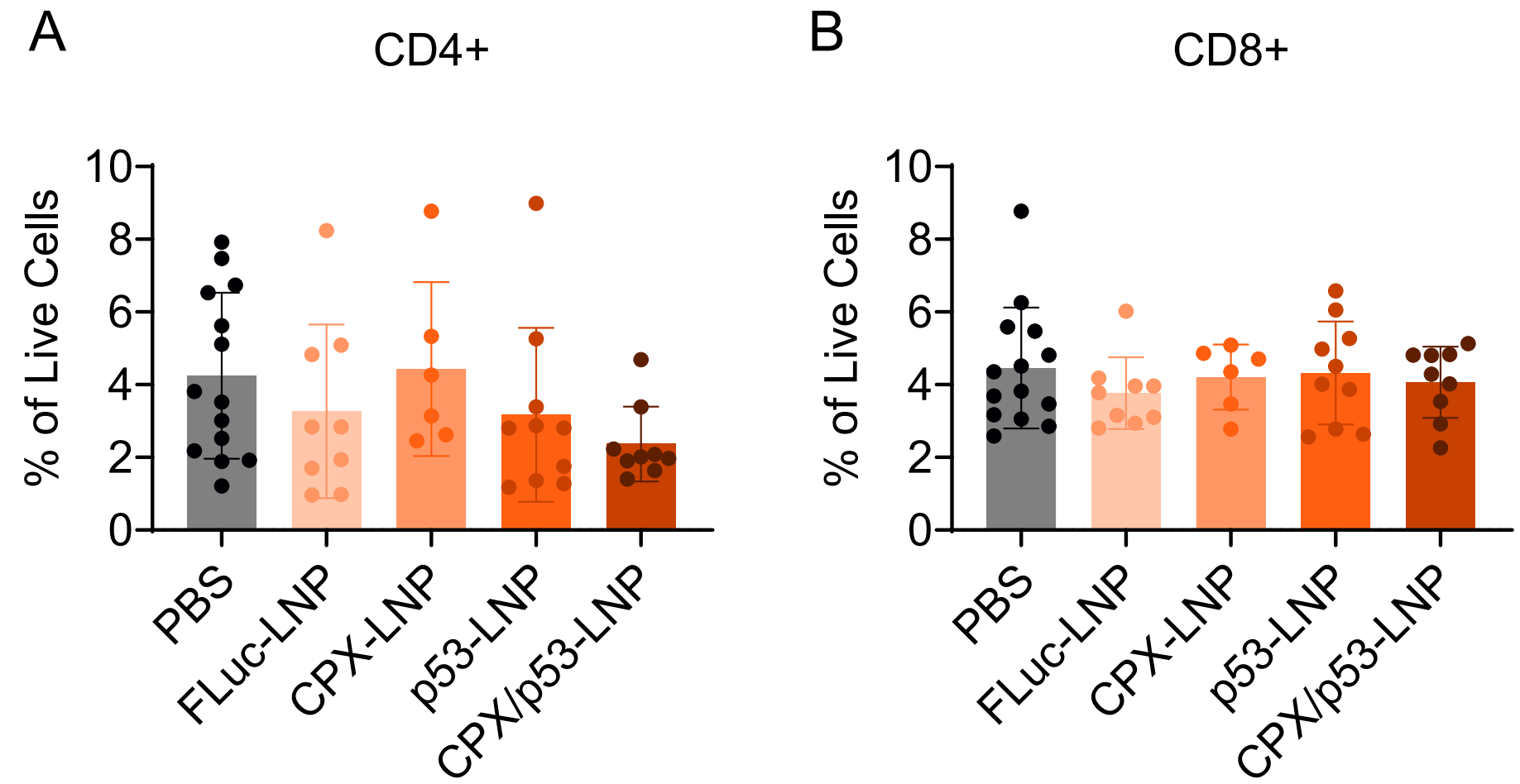

Supplementary Figure 14. Tumor-infiltrating CD4+ and CD8+ T cells in MOC-2 syngeneic model. A–B, E10i-494 LNPs encapsulating firefly luciferase (FLuc-LNP), 50 mol% CPX (CPX-LNP), murine p53 mRNA (p53-LNP), 50 mol% CPX and murine p53 mRNA (CPX/p53-LNP), and PBS were injected three times over four days intratumorally into C57BL/6J mice inoculated with MOC-2 cells. After 48 h from the third dose, tumors were dissected and analyzed via flow cytometry to examine **(A)** CD4+ and **(B)** CD8+ cells, gated from all live cells. Percentage is reported as mean ± SD of n = 14 for PBS, n = 10 for p53-LNP, n = 9 for FLuc-LNP and CPX/p53-LNP, and n = 6 for CPX-LNP. One-way ANOVA with *post hoc* Holm–Šídák correction for multiple comparisons was used to compare formulations. No comparisons reached significance.
